# Application of genomic tools to study and potentially improve the upper thermal tolerance of farmed Atlantic salmon (*Salmo salar*)

**DOI:** 10.1186/s12864-025-11482-4

**Published:** 2025-03-24

**Authors:** Eric H. Ignatz, Melissa S. Allen, Jennifer R. Hall, Rebeccah M. Sandrelli, Mark D. Fast, Guy M. L. Perry, Matthew L. Rise, A. Kurt Gamperl

**Affiliations:** 1 Department of Ocean Sciences, Memorial University of Newfoundland and Labrador, St. John’s, NL A1C 5S7 Canada; 2 Marine Affairs Program, Dalhousie University, Halifax, NS B3H 4R2 Canada; 3 Center for Aquaculture Technologies, San Diego, CA 92121 USA; 4 Aquatic Research Cluster, Ocean Sciences Centre, CREAIT Network, Memorial University of Newfoundland and Labrador, St. John’s, NL A1C 5S7 Canada; 5 Atlantic Veterinary College, University of Prince Edward Island, CIA 4P3, Charlottetown, PE Canada; 6 AquaBounty Canada, Fortune, PE C0A 2B0 Canada

**Keywords:** Heat tolerance, Growth, Aquaculture, Climate change, GWAS, RNA-Seq

## Abstract

**Background:**

The Atlantic salmon (*Salmo salar*) aquaculture industry must mitigate the impacts of rising ocean temperatures and the increased prevalence/severity of marine heat waves. Therefore, we investigated the genetic architecture and gene expression (transcriptomics) responsible for determining a salmon’s upper thermal tolerance.

**Results:**

A genome-wide association study (GWAS) was conducted using fin clips of salmon from a previous incremental thermal maximum (IT_Max_) challenge (*n* = 251) and the North American 50 K SNP chip. IT_Max_ was a highly polygenic trait with low/moderate heritability (mean SNP-based h^2^ = 0.20 and pedigree-based h^2^ = 0.25). Using data from the same fish, a separate GWAS assessed thermal-unit growth coefficient (TGC). Five significant SNPs were detected on chromosomes three and five, and high heritability estimates were calculated for TGC measured as fish grew from 12 to 20 °C (mean SNP-based h^2^ = 0.62 and pedigree-based h^2^ = 0.64). RNA-seq analyses of liver samples (*n* = 5–6 family^-1^ temperature^-1^) collected from the four most and four least tolerant families at 10 and 20 °C were also used to provide insights into potential mechanisms modulating this species’ thermal tolerance. Between the top and bottom families, 347 and 175 differentially expressed transcripts (FDR-adjusted *p* < 0.01; fold-change ≥|2.0|) were identified at 10 and 20 °C, respectively. GO term enrichment analysis revealed unique responses to elevated temperature between family rankings (e.g., ‘blood coagulation’, ‘sterol metabolic process’ and ‘synaptic growth at neuromuscular junction’). qPCR analyses further confirmed differences pertaining to cholesterol metabolism (*lpl*), inflammation (*epx*, *elf3*, *ccl20*), apoptosis (*htra1b*, *htra2*, *anxa5b*), angiogenesis (*angl4*, *pdgfa*), nervous system processes (*insyn2a*, *kcnj11l*) and heat stress (*serpinh1b-1*, *serpinh1b-2*). Three differentially expressed transcripts (i.e., *ppp1r9a*, *gal3st1a*, *f5*) were located in close proximity (± 120 kbp) to near-significant SNPs from the GWAS. Interestingly, *ppp1r9a* and *gal3st1a* have putative neurological functions, while *f5* regulates blood coagulation.

**Conclusions:**

These analyses provide several putative biomarkers of upper thermal tolerance in salmon that could prove valuable in helping the industry develop more temperature-tolerant fish. Further, our study supports previous reports that IT_Max_ has low/moderate heritability in this species, and suggests that TGC at elevated temperatures is highly heritable.

**Supplementary Information:**

The online version contains supplementary material available at 10.1186/s12864-025-11482-4.

## Background

Due to anthropogenic-related climate change, sea surface temperatures are quickly on the rise, and this is endangering numerous aquatic species [1]. This is particularly worrisome in the Northwest Atlantic, where ocean temperatures are increasing at rates faster than the global average [2]. This problem is further compounded by marine heatwaves which are becoming more frequent and severe [3, 4]. While some wild fish may be able to change their migratory patterns, depth distribution or seek out thermal refuges [5, 6], Atlantic salmon (*Salmo salar*) grown in net pens are limited in their movements, and thus, are often unable to avoid suboptimal temperatures [7, 8]. Given the challenges that the Australian (Tasmanian) salmon aquaculture industry is facing [8–10], and that a mass mortality event of farmed salmon recently occurred in Newfoundland (Canada) when high summer ocean temperatures coincided with an active sea lice infestation [11], the industry must take action to avoid such catastrophes and ensure animal welfare.

One potential solution is to incorporate genetic selection for improved upper thermal tolerance and performance into existing breeding programs. Genomic selection has already enhanced numerous production-relevant traits in cultured fishes such as growth, disease resistance and fillet quality [12]. Work has also been conducted to estimate the heritability of the critical thermal maximum (CT_Max_) of Atlantic salmon [13, 14], rainbow trout (*Oncorhynchus mykiss*; [15, 16]), zebrafish (*Danio rerio*; [17]), killifish (*Heterandria formosa*; [18]) and mosquitofish (*Gambusia holbrooki*; [19]). However, CT_Max_ trials involve rapid increases in temperature until the loss of fish equilibrium [20], which rarely reflect ecologically- or industrially-relevant scenarios or traits. Thus, a newer approach has been developed to assess the survival of species like Atlantic salmon that experience more gradual changes in temperature in the natural environment and/or in net pens. The incremental thermal maximum (IT_Max_) method estimates that the average upper thermal tolerance of Atlantic salmon is between 22.8 and 25.3 °C [21–25]. In addition, the heritability of this metric (h^2^ = 0.16–0.40) has recently been estimated for this species [13, 26]. However, less is known about the transcriptional mechanisms underlying IT_Max_, and how these data relate to the genetic architecture that regulates upper thermal tolerance in Atlantic salmon.

Further, it will be important for aquaculture producers to understand how selecting for IT_Max_ in a breeding program can potentially impact other commercially relevant traits like growth. For decades, improving growth rates has been a primary focus of Atlantic salmon breeding programs as genetic gains are high per generation [27] and enhancement leads to direct economic benefits [28]. However, active selection for a particular trait can have trade-offs that detrimentally impact another phenotype of interest [29]. Genetic compensation can occur if conserved molecular pathways are shared between traits, and energy is diverted to one outcome over another [30]. Yet, some salmonid studies have shown positive genetic correlations between growth and other commercially relevant traits like disease resistance and survival during grow-out [31, 32]. Given that Atlantic salmon in cage-culture are exposed to seasonal changes in temperature, it is also important to standardize growth rate measurements and predict performance as temperature increases during the transition to summer. As thermal-unit growth coefficient (TGC) takes temperature into account in its calculation [33, 34], it will be important to assess its relationship to IT_Max_ to help inform breeding strategies.

From a mechanistic perspective, several studies have examined the impact of temperature change/stress on the hepatic transcript expression of Atlantic salmon [24, 35–39]. Thus, the liver is a useful tissue to examine because of its multi-faceted role in nutrient metabolism, endocrine signaling, growth and immunity [40]. While the induction of heat shock protein (*hsp*) genes is recognized as an important component of the heat stress response in teleosts [41], the effects of temperature changes are often more complex. Exposure to high temperatures for acute or prolonged periods generally leads to a robust stress response in salmon that dysregulates a myriad of metabolic pathways and stimulates a broad spectrum of cellular defense processes [35, 39]. For example, a microarray study that compared Atlantic salmon slowly raised (1 °C week^-1^) to 20 °C from the control temperature of 12 °C identified 1900 differentially expressed probes between normoxic (~ 100% air saturation) treatments [35]. Cellular and oxidative stress, immune, and apoptotic pathways were significantly upregulated at the higher temperature, while probes linked to proteolysis, catabolism and cellular metabolism were downregulated [35]. However, less is known regarding the mechanisms that determine a salmon’s ability to withstand and survive at higher temperatures for longer durations. The oyster aquaculture industry is further ahead in this research [42, 43] and its application of genomic-assisted selection to improve upper thermal tolerance [44–46]. Therefore, it is important that the Atlantic salmon aquaculture industry employ similar tools to enhance our understanding of what regulates IT_Max_ and how to improve it.

Therefore, we took an integrative approach to investigate this question, and paired a genome-wide association study (GWAS) with RNA-sequencing (RNA-seq) results to expand from a past experiment that found significant differences in IT_Max_ among 20 Atlantic salmon families, with average upper thermal tolerance ranging from 23.3 to 25.0 °C [23]. Combining individual phenotypes in GWAS and RNA-seq analyses can greatly increase the robustness of the analysis, and over the past decade, has quickly become a standard approach in terrestrial plant/animal breeding and human medical research [e.g., 47–49]. With a special interest in one of the most commercially important traits, a separate GWAS was also performed to examine TGC and its relationship to IT_Max_. Overall, the current study will be of great benefit to Atlantic salmon producers as it provides them with critical information that can inform breeding programs on how to manage the negative effects of climate change, and helps to ensure a sustainable future for the industry.

## Methods

### Experimental animals & rearing conditions

A full description of the animals and experimental procedures involved in this study has been previously published [23]. To summarize, passive integrated transponder (PIT)-tagged conventional mixed-sex, diploid, Atlantic salmon from 20 families of St. John River origin were transported from AquaBounty Canada (PE, Canada) to the Ocean Sciences Centre (Memorial University of Newfoundland and Labrador). Fish from these families were the same age and there was no prior knowledge of their thermal tolerance. The cross structure of the families can be found in Table 1 of Ignatz et al. [23]. The salmon were initially anesthetized (0.2 g L^-1^ AquaLife TMS; Syndel Laboratories Ltd, Nanaimo, BC, Canada), weighed and distributed amongst ten 2.2 m^3^ tanks (~ 6 fish family^-1^ tank^-1^). They were then acclimated to 12 °C for two weeks. At the end of this acclimation period, the fish were anesthetized (0.2 g L^-1^ TMS) and measured for weight and fork length. Additionally, floy tags (Floy Tag & Manufacturing, Inc., Seattle, WA, USA) were inserted into the dorsal muscle of two pre-selected fish family^-1^ tank^-1^ (10 tanks total) to identify salmon that would be sampled later. Based on similar published methodology [50–52], the goal was to eventually sample fish representative of the range of body weights within each family, but exclude the largest and smallest fish from each family. Correspondingly, the fish that were tagged fell within ± 1 standard deviation of their family’s mean weight (determined from the first weight assessment two weeks prior), alternating between every other fish in descending order of body weight. In summary, 20 fish per family were tagged (10 fish in the ‘warm’ treatment and 10 fish in the ‘control’ treatment).

After this procedure, the warm treatment (5 tanks) was exposed to an IT_Max_ challenge at a rate of + 0.2 °C day^-1^. This rate of increase matched previous IT_Max_ experiments [24, 25], and mimicked conditions that farmed salmon would encounter during a Newfoundland summer in sea cages [7, 53]. Conversely, the control treatment (5 tanks) had their temperature lowered to 10 °C (-0.2 °C day^-1^) and remained at this temperature for the duration of the trial. This was done to avoid the development of dermal ulcers in fish kept at temperatures from 12 to 14 °C [24]. When the warm treatment reached 20 °C, another assessment was performed wherein fish from both temperature groups were anesthetized (0.2 g L^-1^ TMS) and weight and fork length were recorded. The previously tagged fish were also sampled at this experimental stage (see below for more details). The temperature in the warm treatment tanks was then increased at 0.2 °C day^-1^ until all remaining non-floy-tagged salmon became moribund (i.e., lost equilibrium) or succumbed, with temperature recorded at this stage for each fish as their IT_Max_. Adipose fin clips were collected from each salmon at their IT_Max_ and preserved in 96% ethanol for subsequent genotyping analyses.

### Sample collection & selection for sequencing

At the 20 °C assessment point, floy-tagged fish were sampled across all 20 families from the warm (20 °C) and control (10 °C) treatments. Fish were taken off feed at least 24 h in advance of sampling and were euthanized by an overdose of anesthetic (0.4 g L^-1^ TMS). Prior to sampling tissue, body weight, fork length, sex and state of sexual maturity were recorded for individual fish. Using standard aseptic techniques, the liver was removed and weighed after the gallbladder was carefully detached. Then, the posterior-most portion of the liver was collected, placed into a 1.5 mL RNase-free tube, quickly flash-frozen in liquid nitrogen, and stored at -80 °C. Although ten fish per family were initially tagged in each temperature group, some of the tags fell off during the experiment and some fish with tags were removed from the study after developing sores, reducing the number of fish that could be sampled. While 5–10 liver samples per family were collected at both 10 and 20 °C, ultimately, only 5–6 samples were chosen from four top and four bottom thermally tolerant families (based on IT_Max_ results; [23]) at each temperature for the RNA-seq experiment. These are the same families identified and chosen for the CT_Max_ experiments performed in Ignatz et al. [23] (i.e., top families– F19, F20, F18, F4; bottom families– F1, F2, F6, F5). With the exception of F2, F4 and F6 at 20 °C (*n* = 5), all other family/temperature combinations contributed six samples for RNA-seq. An attempt was made to balance the male: female ratio (i.e., three males, three females per family at each temperature) during sample selection, but as sex was unknown before sampling, this was not always possible. Where more than six fish had been sampled for a given family, a weight range was calculated (family mean ± 1 standard deviation) to narrow down the most representative samples. If all fish still fit within this weight range, samples were randomly selected for inclusion in the study. Ultimately, a total of 93 samples were chosen for RNA-seq (see Supplemental Table S1).

### RNA extraction and purification

Liver samples were homogenized with stainless steel beads (5 mm; QIAGEN, Mississauga, ON, Canada) in TRIzol^®^ (Invitrogen/Life Technologies, Burlington, ON, Canada), and then centrifuged through QIAGEN QIAshredder columns. RNA extractions were then performed according to the manufacturer’s instructions, with a second extraction completed using the phenol-chloroform phase separation method described in Xu et al. [54]. Extracted RNA (25 µg total RNA per sample) was then DNase I-treated (6.8 Kunitz units; QIAGEN RNase-Free DNase Set) and column-purified (QIAGEN RNeasy Mini Kit) following the manufacturer’s instructions. RNA integrity (sharp 18 S and 28 S ribosomal RNA bands) was confirmed via 1.0% agarose gel electrophoresis and RNA purity (A260/230 and A260/230 ratios were all ≥ 2.00 and 1.80, respectively) was also verified through NanoDrop spectrophotometry. The subsets of each sample that were later shipped to Canada’s Michael Smith Genome Sciences Centre at the BC Cancer Research Institute (Vancouver, BC, Canada) were assessed using a Caliper LabChIP GX Touch HT (Model #: CLS137031; Hopkinton, MA, USA) and all had RNA Quality Scores (RQS) of ≥ 7.3 (average of 8.8).

### Vitellogenin Pre-Screening

During sampling, fish were visually characterized as being sexually immature, maturing or mature. Sexual maturation in Atlantic salmon is known to drive biological changes brought on through transcriptional regulation that can ultimately influence fitness traits like growth and survival [55]. Sexual maturation is also hypothesized to negatively impact the survival of salmonids exposed to high temperatures [56], with sex-specific outcomes [57, 58]. Therefore, to assess potential quantitative differences in sexual maturation between the sexes, families and temperature groups before sequencing, expression of *vitellogenin* (*vtg*) was measured using real-time quantitative polymerase chain reaction (qPCR). Based on the results of Andersen et al. [59], *vtgAsa1* was chosen for screening as it was the most responsive paralogue in Atlantic salmon liver during female sexual maturation. cDNA was synthesized from 1 µg of DNase-treated, column-purified RNA using random primers (250 ng; Invitrogen/Thermo Fisher Scientific; Mississauga, ON, Canada), dNTPs (0.5 mM final concentration; Invitrogen/Thermo Fisher Scientific) and M-MLV reverse transcriptase (200 U; Invitrogen/Thermo Fisher Scientific) in 1X first strand buffer and DTT (10 mM final concentration) following the manufacturer’s instructions. In addition to *vtgAsa1*, three normalizer transcripts (i.e., *ef1a*, *pabpc1* and *rpl32*) were assessed based on previous knowledge of their stability in Atlantic salmon liver [24, 37, 38] with primers taken from past research [54, 60, 61]. Each qPCR reaction consisted of: 6.5 µL of 2X Power SYBR Green PCR Master Mix (Applied Biosystems; Thermo Fisher Scientific), 1.46 µL of nuclease-free water, 0.52 µL (50 nM) of both the forward and reverse primers and 4 µL of cDNA (diluted 1:40) representing 5 ng of input total RNA. Amplifications were performed with a ViiA7 Real-Time PCR system (Applied Biosystems) using a real-time analysis program consisting of 1 cycle of 50 °C for 2 min, 1 cycle of 95 °C for 10 min and 40 cycles of 95 °C for 15 s then 60 °C for 1 min with fluorescence detection at the end of each 60 °C step, and was followed by dissociation curve analysis. Samples were run in triplicate alongside no-template controls (NTCs) for each transcript. Female and male samples were run on separate plates, with a cDNA pool created using equal concentrations of RNA from female samples included on both qPCR plates to serve as a linker (i.e., used to ensure there was no plate-to-plate variability). In addition, an equimolar pool of cDNA from all samples was used to test amplification efficiencies. Standard curves were generated using five-point serial dilutions (1:3) from 10 ng of input RNA and included NTCs. Primer efficiencies met standard quality parameters [62] for linearity (r^2^ ≥ 0.998) and ranged between 94 and 104% (Table 1). Testing also confirmed that single products were amplified (dissociation curve analysis) with no evidence of primer-dimers and no contamination present in the NTCs.

Raw cycle threshold (C_T_) data were imported into qbase+ (Biogazelle; Ghent, Belgium) [63], where technical replicates outside of ± 0.5 C_T_ from two close replicates were removed. Analysis through geNorm identified *ef1a* and *pabpc1* as the most stable normalizers tested (mean geNorm M value and coefficient of variation of 0.207 and 0.072, respectively). Normalized relative quantities (NRQ) were calculated with amplification efficiencies incorporated in qbase + when analysing the female and male samples separately. Calibrated normalized relative quantities (CNRQ) were calculated in the same way, but also incorporated the C_T_ values of linker samples across plates when evaluating female and male samples together. In both cases, the sample with the lowest normalized expression level was assigned a (C)NRQ value of 1.0. Expression of *vtgAsa1* was not detected in one female (top family F20 at 10 °C) and one male (bottom family F5 at 20 °C).

**Table 1:** qPCR primers

Gene Name (GenBank Accession Number)	Nucleotide sequence (5’-3’)	Amplification Efficiency (%)	*r* ^2^	Amplicon Size (bp)	Source
*angiopoietin-related protein 4* (*angl4*) (NM_001139941.1)	F: CAACCCTGACTCTGTGCCTG	85.1	0.992	126	This study
	R: TCACATTCACGTCGTCCCAC				
*annexin A5b* (*anxa5b*) (NM_001141036.1)	F: CAGTGTGAGAGCCAGTGGAA	89.5	0.998	132	Xu et al. [54]
	R: TGTCTCTGGCTGTTGCTACG				
*C-C motif chemokine 20* (*ccl20*) (XM_014159437.2)	F: AGGCTACACCTTCCAGGACA	98.3	0.996	119	This study
	R: GCAGTACCCTCTTGGTCCAG				
*E74-like factor 3* (*elf3*) (XM_014131843.2)	F: CCAGAGCCTGGTGGAACTAA	98.4	0.994	90	This study
	R: GGGAAGTCTGGGAACTCCTC				
*eosinophil peroxidase-like* (*epx*) (XM_014147274.2)	F: CACCCATCAAGTGCAACAAC	99.1	0.996	100	This study
	R: TCCTGGTCCGAATACTCCTG				
*HtrA serine peptidase 1b* (*htra1b*) (XM_014158365.2)	F: ATGATGACTCTCACACCAATGC	97.8	0.996	104	Caballero-Solares et al. [80]
	R: GTTTTTGGGATGACCTCGATT				
*serine protease HTRA2*,* mitochondrial-like* (*htra2*) (XM_014180216.2)	F: CTTCCTGAACCAAGCCTCTG	94.7	0.991	128	This study
	R: GATCCCTCAGCTTCAACTCG				
*inhibitory synaptic factor 2a* (*insyn2a*) (XM_014154695.2)	F: TGCCAACCCTTCTACCAAGC	110.3	0.981	116	This study
	R: CTAGACCTTCCACCTGCGTC				
*potassium inwardly rectifying channel subfamily J member 11*,* like* (*kcnj11l*) (BT059682.1)	F: GTATGCAGCACAGCCAGAGA	107.3	0.989	115	This study
	R: TCCCAGACCTTCCACCTCAA				
*lipoprotein lipase* (*lpl*) (XM_014148677.2)	F: GGTGTCAGCGCTGTATGAGA	101.3	0.981	90	This study
	R: GCGGCTACAGGATACTGGTT				
*platelet-derived growth factor subunit A-like* (*pdgfa*) (XM_045708160.1)	F: TCATCTGGCCTCCCTGTGTA	89.9	0.991	114	This study
	R: CCACCTTAGCCACCTTGACA				
*serine peptidase inhibitor*,* clade H*,* member 1a-1* (*serpinh1a-1*; alias *hsp47a-1*) (OQ814177)	F: GGGCGAGAAGATGAGAGATG	110.0	0.968	199	Ignatz et al. [77]
	R: GCACGAATGTTGGCACATAG				
*serine peptidase inhibitor*,* clade H*,* member 1a-2* (*serpinh1a-2*; alias *hsp47a-2*) (OQ814179)	F: CCAATGTCTTCCATGCCTCT	93.8^a^	-	94	Ignatz et al. [77]
	R: TTGGGGTTCTTCAGCTTGTC				
*serine peptidase inhibitor*,* clade H*,* member 1b-1* (*serpinh1b-1*; alias *hsp47b-1*) (OQ814181)	F: CCAAAGTCAGCATGGAGGTT	101.3	0.994	151	Ignatz et al. [77]
	R: ATTGAAGAGGCGTGGAACAC				
*serine peptidase inhibitor*,* clade H*,* member 1b-2* (*serpinh1b-2*; alias *hsp47b-2*) (OQ814182)	F: CTCATCATGCCCTACCACCT	103.3	0.995	188	Ignatz et al. [77]
	R: TCCACAGCTTCAGTCACACC				
*vitellogenin* (*vtgAsa1*) (XM_014168660)	F: TGAAGGACTTTGGTCTGGCTTACACA	103.4	0.999	50	Andersen et al. [59]
	R: CTGCTGGCACTCTACACACTTC				
*elongation factor 1 alpha* (*ef1a*) (NM_001141909)^b^	F: GTGGAGACTGGAACCCTGAA	99.6	0.998	155	Jones et al. [60]
	R: CTTGACGGACACGTTCTTGA				
*polyadenylate-binding protein 1* (*pabpc1*) (EG908498)^b^	F: TGACCGTCTCGGGTTTTTAG	94.0	0.999	108	Xu et al. [54]
	R: CCAAGGTGGATGAAGCTGTT				
*60 S ribosomal protein 32* (*rpl32*) (BT043656)^c^	F: AGGCGGTTTAAGGGTCAGAT	96.8	0.998	119	Xue et al. [61]
	R: TCGAGCTCCTTGATGTTGTG				

^a^ Expression was too low to calculate an amplification efficiency in the current study. This value comes from Ignatz et al. [77] where an efficiency was calculated from other Atlantic salmon liver cDNA

^b^ Normalizer gene chosen for this study

^c^ Normalizer gene tested, but ultimately not chosen for this study

### Sequencing, read alignment and annotation

Library construction, sequencing and bioinformatics analyses were completed by Canada’s Michael Smith Genome Sciences Centre at the BC Cancer Research Institute. A total of 93 stranded paired-end 150 bp libraries were created from the RNA samples collected from the top and bottom thermally tolerant families at 10 and 20 °C using an Illumina NovaSeq 6000 platform (v.1.5 chemistry) targeting 50 M reads per library (see Supplemental File S1 for more details). Sequencing and alignment metrics of each biological replicate RNA-seq library can be found in Supplemental Table S1. Raw sequencing data is available through the NCBI Sequence Read Archive (SRA) database under BioProject PRJNA912749. Sequenced transcriptome libraries were screened using BioBloom filters [64] to remove any reagent, bacterial/viral and/or non-target species contamination. Each library was also reviewed to determine the number of reads matching the target species (i.e., *Salmo salar*), as well as assess rRNA and mitochondrial content. Plasmid spike-in and single nucleotide polymorphism (SNP) concordance were used to check for potential sample swaps and low GC content, and transcript vs. intergenic ratios were assessed to identify any cases with poor nucleic quality. All 93 samples passed Canada’s Michael Smith Genome Sciences Centre’s internal quality control thresholds. STAR (v.2.5.2b) [65] was used to align the RNA-Seq data to the genome reference (*Salmo salar* assembly Ssal v3.1 taken from NCBI– Accession GCA_905237065.2). RSEM (v.1.3.0) then quantified transcript expression, as well as estimated their abundance as fragments per kilobase of exon per million fragments mapped (FPKM) and transcripts per million (TPM) values [66]. Ensembl gene annotation (v.106) created gene transfer format (GTF) files to build the RSEM reference. The transcript reads were used as input to Trinity (v.2.14.0) with no depth filter applied to assemble/gather the contig sequences [67]. BLAST was then used to annotate the contigs that mapped to each transcript with both *Salmo salar* (BLASTn) and putative *Homo sapiens* (BLASTx) orthologue accession identifiers [Expect (E) value cut-off < 1e-5]. Putative human orthologues from NCBI’s RefSeq protein database (GRCh38.p14) annotated 84.0% of the transcriptome, and were used for downstream analyses (i.e., GOrilla and ClueGO) as most of the *Salmo salar* genome is not reliably functionally annotated.

### Differential expression, GO term enrichment and pathway analyses

Using raw read counts, DESeq2 (v.1.28.1; [68]) was used to determine differentially expressed transcripts between four comparisons with regards to family ranking and temperature treatment: (1) top vs. bottom families at 10 °C (*n* = 24 for each group); (2) top vs. bottom families at 20 °C (*n* = 23 & 22, respectively); (3) top families at 20 vs. 10 °C (*n* = 23 & 24, respectively); and (4) bottom families at 20 vs. 10 °C (*n* = 22 & 24, respectively). For each comparison, transcripts were filtered out if the median raw count for both sets of libraries was less than 25. Transcripts were considered differentially expressed if their false discovery rate (FDR)-adjusted *p*-value was below 0.01 and the fold-change difference between groups was ≥|2.0|. To avoid sexual maturity as a potential confounding factor, DETs that were hierarchically clustered and significantly correlated (*p* < 0.05) with *vtgAsa1* expression were removed from the two comparisons between the top and bottom families at 10 and 20 °C (see Supplemental Table S2 for the full list). Hierarchical clustering (heatmaply package) and correlation (corrplot package) analyses were conducted in R (v.4.3.0). Comparisons between DET lists were made with Venny (v.2.1; [69]). Standardized TPM values (calculated in the same manner as the standardized FPKM values in Ignatz et al. [38]) of the DETs from the comparisons of the top and bottom families were used as input in the principal component analysis (PCA; factoextra R package). One-way ANOVAs assessed differences (*p* < 0.05) within principal component axis (PC) scores among treatments.

Using DAVID (v.2024q2; [70, 71]), gene symbol and Entrez identifications were obtained by converting the RefSeq accessions of putative human orthologues from the re-annotated transcriptome. These identifiers were then used as input for GOrilla [72, 73] and ClueGO (v.2.5.9; [74]) analyses, respectively. Unranked target (*Homo sapiens* annotated DET list) and background (entire *Homo sapiens* annotated transcriptome from the current study; Supplemental Table S3) transcript lists were used for both platforms. The study-specific background list was chosen as it reflects the most accurate representation of the genes that were expressed in these salmon [75, 76]. In GOrilla, duplicates were included in the output and GO terms were considered significantly enriched if FDR-adjusted *p*-values fell below 0.05. However, GO terms with unadjusted *p*-values < 0.05 were included in some cases for exploratory analyses. Based on the visual output of the GOrilla platform, the hierarchical level of specific GO terms was assessed manually. ClueGO was run on Cytoscape (v.3.10.2) to illustrate enriched GO terms (v.25.05.2022) and KEGG pathways (v.25.05.2022) amongst annotated up- and downregulated DETs. Two-sided hypergeometric tests were performed with a Bonferroni step-down *p*-value adjustment. A kappa statistic threshold of 0.4 and a medium specificity level were used to design the networks while only including terms with *p* < 0.05.

### qPCR validation

Eleven transcripts of interest (TOIs) were chosen for qPCR validation of the RNA-seq results based on their presence in the DET lists comparing the top and bottom thermally tolerant families at both 10 and 20 °C (i.e., *anxa5b*, *ccl20*, *elf3*, *epx*, *htra1b*, *htra2*, *kcnj11l*, *lpl* and *pdgfa*) or just at 20 °C (i.e., *angl4* and *insyn2a*). These specific transcripts were targeted as they have putative functions among a wide range of biological pathways, including cholesterol metabolism (*lpl*), inflammation (*epx*, *elf3*, *ccl20*), apoptosis (*htra1b*, *htra2*, *anxa5b*), angiogenesis (*angl4*, *pdgfa*) and nervous system processes (*insyn2a*, *kcnj11l*). They also had no correlation (*p* > 0.05) in expression to any of the *vitellogenin* transcripts originally in the DET lists. In addition, the four paralogues of *serpinh1* were assessed via qPCR given their putative central role in mediating thermal stress in salmonids [35, 36, 77–79]. Several primer pairs came from past studies [55, 77, 80]. For primers new to this study, a BLASTn search of the non-redundant nucleotide (nr/nt) sequence database of NCBI [*Salmo salar* (taxid: 8030) sequences only] was performed using the RNA-seq generated sequence to identify annotated paralogues/isoforms; if present, the primers were designed in an area with ≥ 3 bp difference between them to ensure specificity. Primers were designed with a melting temperature (T_m_) of 60 °C mostly using Primer3 [81–83]. However, for some gene paralogues/isoforms, they were hand-designed in paralogue/isoform-specific areas to ensure specificity.

All primer pairs were (re-)subjected to quality control testing using samples from the current study. Briefly, 350 ng of 32 representative (i.e., 8 families x 2 sexes x 2 temperatures) individual column-purified RNA samples were combined to generate an RNA pool that was then utilized in the cDNA synthesis. To calculate amplification efficiencies ( [62]; Table 1), standard curves were generated using a 5-point 1:3 dilution series starting with cDNA representing 10 ng of input total RNA. Each primer pair was also tested to ensure that a single product was amplified (dissociation curve analysis) and that no primer-dimer was present in the NTC control. With the exception of *ef1a*, which had a slight shoulder to the left of the primary peak, all amplicons had sharp, single peaks in the melt curves. Finally, amplicons were electrophoretically separated on 2.0% agarose gels and compared with a 1 kb plus ladder (Invitrogen) to verify that the correct size fragment was being amplified.

The qPCR validation was conducted according to MIQE guidelines [84]. The same approaches as described above under the *vitellogenin* pre-screening were used for cDNA synthesis, including using the same RNA that was sent for sequencing, and for PCR amplifications with the exception that a QuantStudio 6 Flex Real Time PCR system (Applied Biosystems) was used. cDNA corresponding to 5 ng of input total RNA was used as template in the PCR reactions. For each sample, the TOI and endogenous controls were tested in triplicate on the same plate and an NTC was included. With this design, expression levels were assessed across four plates, so a plate linker sample was also present. The CNRQ of each transcript was determined using the QuantStudio Real Time PCR Software (v.1.7.2; Applied Biosystems) relative quantification study application, with normalization to both *ef1a* and *pabpc1* transcript levels, and with amplification efficiencies incorporated. Like in the pre-screening, *ef1a* and *pabpc1* were stable normalizers, with < 0.5 C_T_ difference in averages between treatments considering family ranking, temperature and sex. For each TOI, the sample with the lowest normalized expression (mRNA) level was set as the calibrator sample (i.e., assigned a CNRQ value = 1.0). T-tests in R were used to compare family rankings at a particular temperature and between temperatures within a family ranking.

### Genotyping

The fin clip samples collected from salmon at their IT_Max_ (*n* = 265) and blood samples from all 33 parents were shipped to the Center for Aquaculture Technologies Canada (Souris, PE, Canada) for processing. Following DNA extraction, samples were genotyped using a high-density Affymetrix 50 K SNP array designed specifically for North American strain Atlantic salmon [85]. Parents were included in duplicate on the array. Raw Affymetrix format output data (CEL files) were generated and used to produce calibrated/cluster-called genotypes for each locus across all samples in Axiom Analysis Suite after filtering using the standard SNPolisher pipeline with the recommended dishQC metrics (Thermo Fisher Scientific). An individual call rate filter for genotype missing-ness no greater than 20% was applied to the data and the genome-wide associated study (GWAS) analyses proceeded with a total of 251 animals. SNP data were trimmed for quality, and after removing SNPs with call rates below 80% and minor allele frequencies less than 2%, 46,380 SNPs remained.

### Genome-Wide association studies

GWAS analyses were conducted for TGC and IT_Max_ using Genome-wide Complex Trait Analysis (GCTA; [86]) software. TGC was calculated from weight gain measurements of individual fish from 12 °C up to 20 °C (i.e., during the 36 days that temperature was raised in the warm treatment) during the incremental thermal challenge using the following formula [33, 34]:

$$\:\text{T}\text{G}\text{C}=\left(\frac{\text{W}_{f}^{1/3}\text{ - }\text{}\text{W}_{i}^{1/3}}{{\sum\:}_{\text{i}=1}^{\text{n}}\text{T}_{i}}\right)\:\times\:1000$$

where W_f_ and W_i_ are the final and initial fish body weights (in g), respectively, n is the number of days since W_i_, and T_i_ is mean daily water temperature (in °C). As there was no relationship between TGC and initial fish weight (r^2^ < 0.01; *p* = 0.168), starting weight was not included as a covariate as it was not believed to bias the results. Tank was included as a covariate in the IT_Max_ model, but not for TGC, as it its presence was not found to affect the results (*p* > 0.05). No corrections were applied to the phenotypic data, as there were not deviations from normality in the TGC and IT_Max_ datasets based on the QQ plots. The family averages of TGC and IT_Max_ are provided in Tables 5 and 6 of Ignatz et al. [23], respectively. Two models were run: (i) mlma using a grm to account for polygenic effects and population structure; and (ii) mlma-loco which was fit with a grm to account for polygenic effects and population structure, but also with markers from the tested SNPs’ chromosomes removed to avoid double counting of variance. Results from the two models were nearly identical, so only the results from the mlma model were used following the general form:

$$\:y\hspace{0.17em}=\hspace{0.17em}a\hspace{0.17em}+\hspace{0.17em}bx\hspace{0.17em}+\hspace{0.17em}g\hspace{0.17em}+\hspace{0.17em}e$$

where y is the phenotype (i.e., IT_Max_ or TGC), a is the mean, b is the additive (i.e., fixed) effect of the candidate SNP to be tested for association, x is the SNP genotype indicator that takes a value of 0, 1 or 2 based on the genotype of the individual, g is the polygenic (i.e., random) effect and e is the residual.

### Heritability

To estimate pedigree and SNP-based heritabilities, the phenotypes of TGC and IT_Max_ were analysed in GCTA based on the methodology proposed by Zaitlen et al. [87], which estimates pedigree-based and SNP-based h^2^ simultaneously in one model using family data following the general model form:

$$\:y\hspace{0.17em}=\hspace{0.17em}X\beta\hspace{0.17em}+\hspace{0.17em}Wu\hspace{0.17em}+\hspace{0.17em}\epsilon$$

where y is an *n* × 1 vector of phenotypes with n as the sample size, X is the covariates, β is a vector of fixed effects, W is a standardized genotype matrix, u is a vector of SNP effects and ε is a vector of residual effects. Genetic correlations were analysed using multivariate linear mixed models. A trait mean and a tank by trait interaction were fit as fixed effects, both found to be significant, while the effect of the family was modelled as random for each trait. The genetic effects, which were treated as random, were fit using a general correlation structure in ASReml-R and assuming variance of genetic effects as the Kronecker product of the variance-covariance matrix (for genotypes across the traits) and the genomic relationship matrix was constructed from the genotypes using the VanRaden approach [88]. Individual residual variances were fit for each trait. The equivalent univariate models were also tested, and produced heritabilities consistent with the multivariate model and the GCTA approach.

### Correlations between RNA-Seq and GWAS analyses

The genomic locations of DETs identified by comparing the top and bottom thermally tolerant families at 10 and 20 °C were used to investigate whether there was any colocalization to SNPs of interest (unadjusted *p*-values < 0.002) from the GWAS analyses for the traits TGC and IT_Max_. DET and SNP locations were first examined to determine if any SNPs fell directly within transcript coordinates, and then the search was expanded outwards to see if SNPs were located within 120 kbp of DETs.

## Results

### Differential expression analyses

Initially, 125.0 ± 8.5 M raw NovaSeq reads were obtained per sample, of which 91.9 ± 0.1% (mean ± SE) were aligned to the reference *Salmo salar* genome (Supplemental Table S1). Using the final read counts, pairwise analyses between thermal tolerance rankings (i.e., top and bottom families) and temperature treatments (i.e., 10 and 20 °C) were conducted using DESeq2. Based on a preliminary assessment of *vtgAsa1* expression, it was determined that while some female salmon were sexually maturing, this likely did not impact their IT_Max_ (Supplemental File S2). However, as several *vitellogenin* transcripts were originally present in the DET lists that compared the top and bottom families (Supplemental Table S2), these, including any transcripts that were hierarchically clustered and significantly correlated (*p* < 0.05) with *vitellogenin* expression, were removed from downstream analyses. Only 8.4% of transcripts (32 out of 379) were removed from the 10 °C comparison and 15.9% of transcripts (33 out of 208) from the 20 °C comparison of the top and bottom families. This precautionary approach was taken to ensure that sexual maturity did not potentially confound the results.

**Fig. 1:**
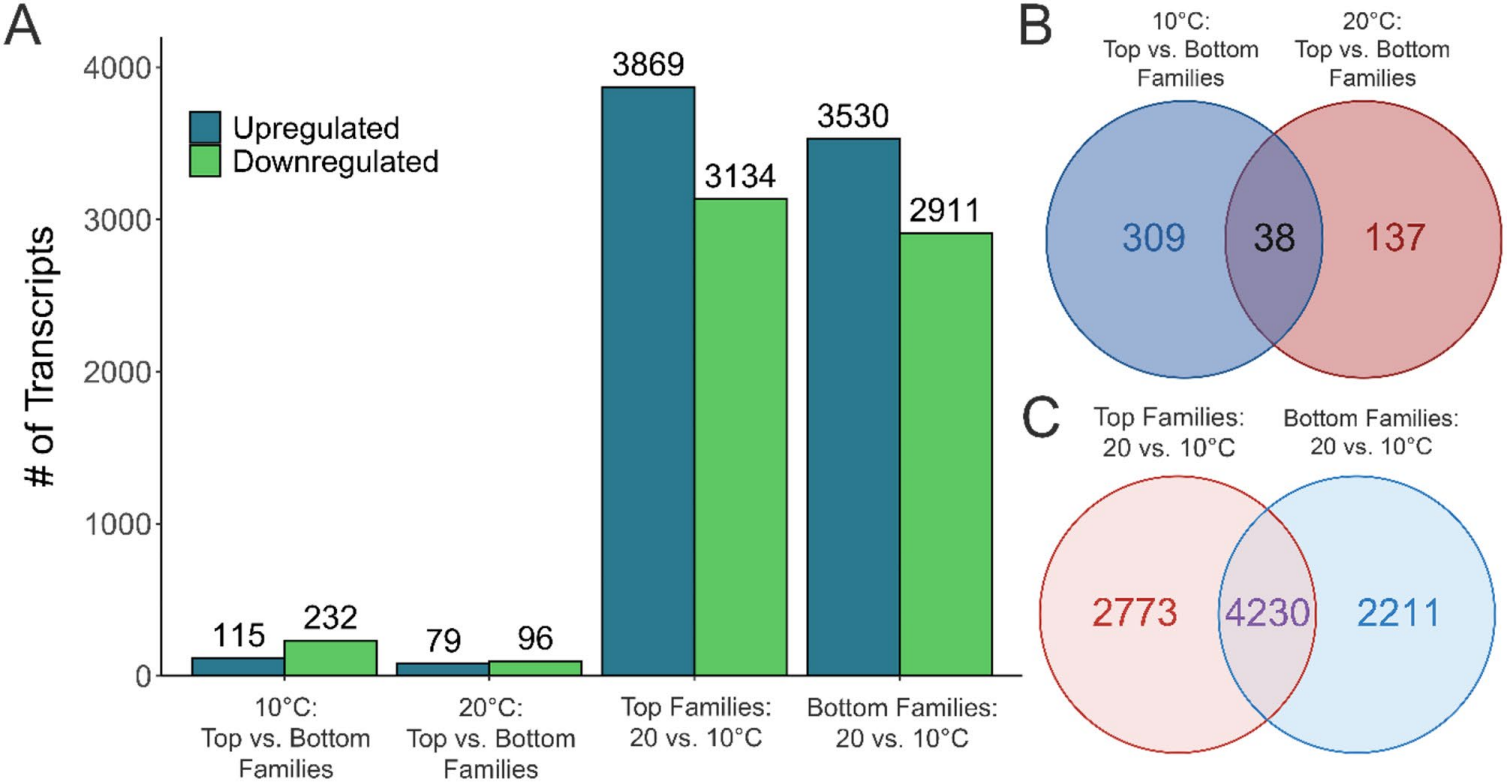
Differential expression between top and bottom thermally tolerant families and at different temperatures. **(A)** The number of up- and downregulated differentially expressed transcripts (DETs) among comparisons identified by DESeq2 analyses (FDR-adjusted *p*-value < 0.01, fold-change ≥|2.0|). **(B)** Venn diagram depicting the number of shared and separate DETs (both up- and downregulated) between the comparisons of the top and bottom families at 10 and 20 °C. **(C)** Venn diagram showing the number of shared and separate DETs between the independent comparisons of temperature treatments within the top and bottom families

Numerous significant DETs (FDR-adjusted *p*-value < 0.01; fold-change ≥|2.0|) were identified in each comparison (Fig. 1A): (1) top vs. bottom families at 10 °C (347 DETs; Supplemental Table S4); (2) top vs. bottom families at 20 °C (175 DETs; Supplemental Table S5); (3) top families at 20 vs. 10 °C (7003 DETs; Supplemental Table S6); and (4) bottom families at 20 vs. 10 °C (6441 DETs; Supplemental Table S7). Within the comparisons at 10 and 20 °C, 38 transcripts were consistently differentially expressed between the top and bottom families (Fig. 1B). While approximately two-thirds of the DETs within each list were shared between the independent temperature-based comparisons of the top and bottom families, each list contained over 2000 transcripts that were unique to that family ranking (Fig. 1C). Collectively, these results indicate that unique expression patterns exist between the top and bottom families at each temperature and in response to elevated temperature.

**Fig. 2:**
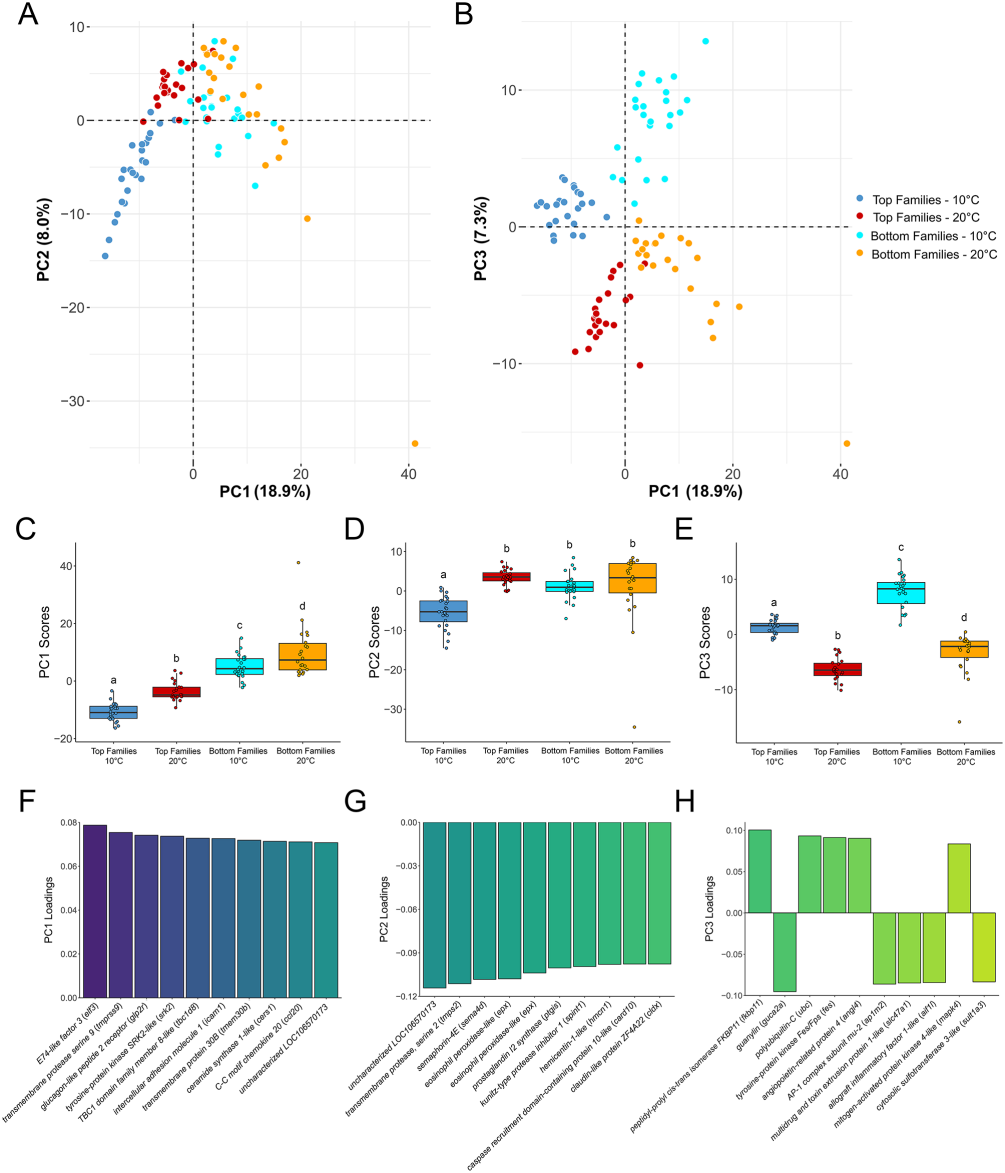
Principal component analysis (PCA) of standardized transcripts per million (TPM) values of the 484 unique differentially expressed transcripts (DETs) identified in Atlantic salmon from the two comparisons of the top and bottom thermally tolerant families at 10 and 20 °C (*n* = 93 in total). **(A)** PCA plot showing the top two principal component axes (i.e., PC1 and PC2) that explain the highest amount of variance in the dataset. **(B)** PCA plot showing PC1 and PC3. **C-E)** Boxplots of PC1, PC2 and PC3 scores from the plots in panels A and B. Dissimilar lowercase letters signify differences among treatments (1-way ANOVA, *p* < 0.05). **F-H)** The ten DETs with the highest loadings on PC1, PC2 and PC3. The DETs are listed in descending order coinciding with the change in colour gradient

To explore the direct differences in transcript expression between the top and bottom families at 10 and 20 °C, expression data of the 484 unique DETs from those comparisons were input into a PCA (Fig. 2A, B). There was significant separation (*p* < 0.05) of each treatment along PC1 and PC3 (Fig. 2C, E), while only the top families at 10 °C separated (*p* < 0.05) from the three other treatments along PC2 (Fig. 2D). Among the top 10 DETs contributing to the variance of each axis (Fig. 2F-H), several were qPCR-validated transcripts (i.e., *elf3*, *ccl20*, *epx* and *angl4*). Interestingly, there were three transcripts encoding transmembrane proteins present in these lists (i.e., *transmembrane protease serine 9*, *transmembrane protein 30B* and *transmembrane protease serine 2*). Overall, these results show distinct expression profiles among each group of families and between temperatures.

### GO term enrichment and pathway analyses

GO term enrichment analyses were performed using the differentially expressed transcript lists (both up- and downregulated DETs) to investigate what functional features differed between the top and bottom families, and how each family ranking responded to an increase in temperature. Comparing the top vs. bottom families at 10 °C, only the ‘extracellular region part’ was significantly enriched (FDR-adjusted *p*-value < 0.05; Supplemental Table S8). However, 31 other GO terms were enriched with unadjusted *p* < 0.05, and served to identify data trends (Supplemental Table S8). Likewise, 35 enriched (unadjusted *p* < 0.05) GO terms were identified when comparing the top vs. bottom families at 20 °C with none meeting the threshold for significance (i.e., FDR-adjusted *p* > 0.05; Supplemental Table S9). Six similar GO terms overlapped between these two lists: ‘extracellular structure organization’, ‘extracellular region part’, ‘extracellular vesicle’, ‘extracellular organelle’, ‘extracellular exosome’ and ‘extracellular space’. Of note, several GO terms were detected at 20 °C related to vitamin D metabolism and growth factors. In contrast, 406 and 314 GO terms were significantly enriched (FDR-adjusted *p*-value < 0.05) when comparing 10 vs. 20 °C in the top and bottom families, respectively (Supplemental Tables S10 and S11). The higher number of enriched GO terms in the top families coincides with the higher number of input DETs (i.e., 7003 vs. 6441 DETs). From the combined number of enriched GO terms, 40.4% (207 terms) were shared between the top and bottom families. Of particular interest and all at the same level of GO organization (level 5), ‘cellular response to endogenous stimulus’, ‘regulation of cellular response to stress’, ‘blood coagulation’, ‘negative regulation of hemostasis’, ‘regulation of signal transduction’, ‘regulation of lipid transport’ and ‘sterol metabolic process’ were all biological processes only enriched in the top families. Conversely, at the same level of GO organization, ‘polysaccharide catabolic process’, ‘lipoprotein metabolic process’, ‘cellular carbohydrate catabolic process’, ‘protein catabolic process’ and ‘synaptic growth at neuromuscular junction’ were all biological processes unique to the bottom families. Thus, these differentially enriched GO terms provide mechanistic insights into how the top and bottom families responded distinctly to an increase in temperature up to 20°C.

**Fig. 3:**
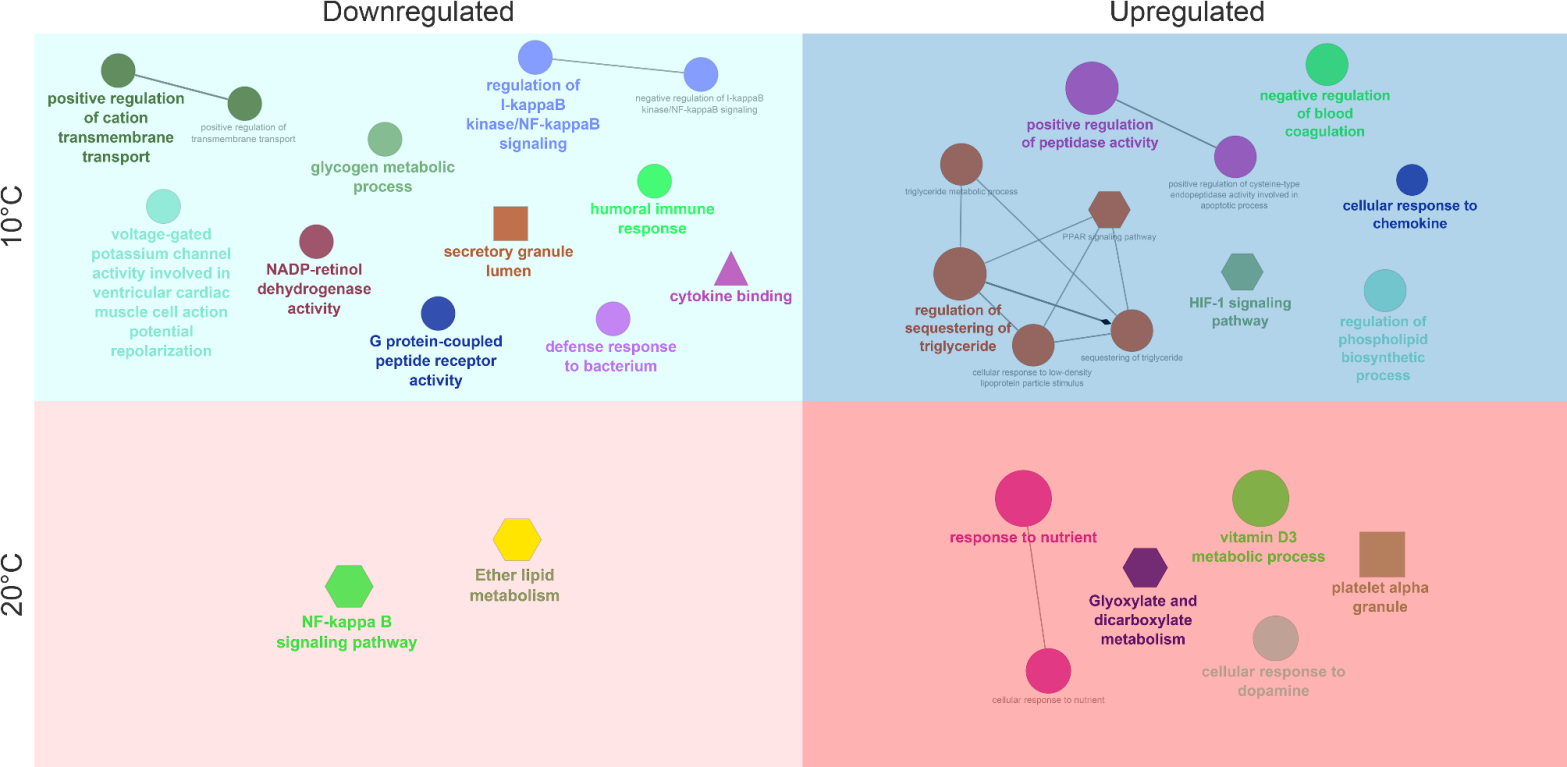
Gene ontology (GO) term enrichment and pathway network analysis of differentially expressed transcripts (DETs) between the top and bottom thermally tolerant families at 10 and 20 °C. Each quadrant represents the up- or downregulated DETs (top relative to bottom families) at a given temperature. In this way, nodes in the light blue and light pink quadrants are enriched terms from downregulated DETs in the top compared to the bottom families at 10 and 20 °C, respectively. Similarly, nodes in the dark blue and dark pink quadrants are enriched terms from upregulated DETs in the top compared to the bottom families at 10 and 20 °C, respectively. The size of the nodes within each independent quadrant indicates the level of significance for that specific term, with larger nodes corresponding to lower *p*-values. Node colours within each quadrant signify related processes that share similar DETs. The shape of the node reflects the database where the term originated (GO Biological Process– circle; GO Molecular Function– triangle; GO Cellular Component– square; KEGG pathway– hexagon)

When organizing the up- and downregulated DETs obtained from comparing the top and bottom families at 10 and 20 °C separately, several significantly enriched GO terms and pathways were identified with ClueGO (Fig. 3). Highlighting a few key features, in the top families at 10 °C, some immune, signaling and transport activities were downregulated. In contrast, lipid, peptidase and HIF-1 signaling pathways, as well as the negative regulation of blood coagulation, were upregulated in the top families compared to the bottom. Only the NF-kappa B signaling pathway and ether lipid metabolism were downregulated at 20 °C in the top families. However, several nutrient/metabolic pathways were upregulated alongside responses to dopamine compared to the bottom families. These pathway analyses help discriminate the direction of transcriptional regulation and further emphasize the distinctive expression patterns between the top and bottom thermally tolerant families.

### qPCR validation

**Fig. 4:**
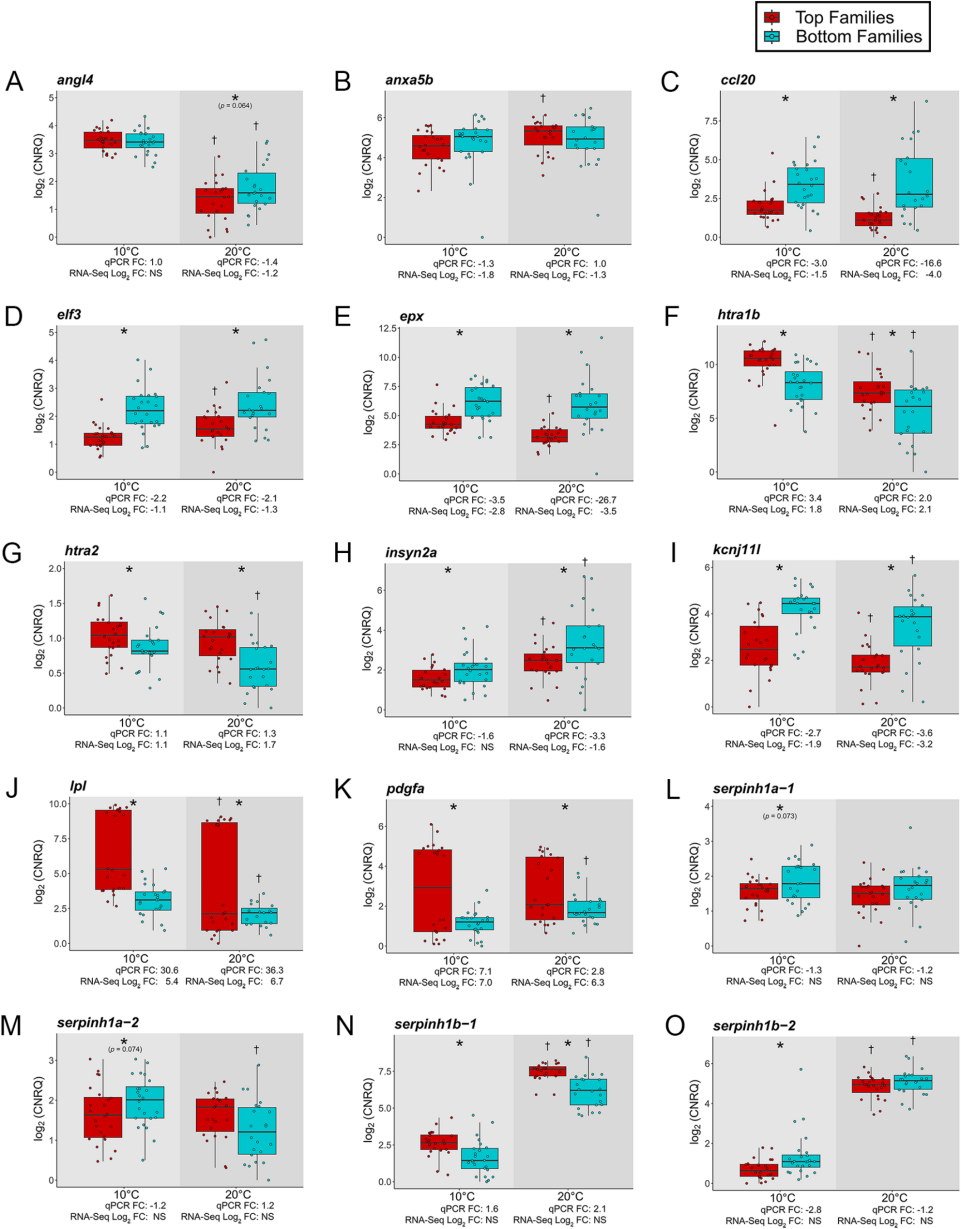
qPCR results for 11 targeted transcripts chosen from the differentially expressed transcript lists comparing the top and bottom thermally tolerant families at 10 (*n* = 24 top; *n* = 24 bottom) and 20 °C (*n* = 23 top; *n* = 22 bottom) in the RNA-seq study, plus 4 paralogues of *serpinh1*. Calibrated normalized relative quantities (CNRQs) were log_2_-transformed. Asterisks (*) denote a significant (*p* < 0.05, unless otherwise noted) difference between top and bottom families within a given temperature treatment. Daggers (†) indicate whether expression differed significantly within a category of families at 20 °C compared to 10 °C. qPCR fold-change (FC) was calculated by dividing the average expression levels of the top families by the bottom families. The log_2_-FC from the RNA-seq study was calculated using DESeq2 and is the average FC where multiple transcripts with the same gene name were differentially expressed. In some cases, comparing the top and bottom families led to a non-significant (NS) result at a specific temperature; this is indicated in the figure as well. Two samples from the top families at 10 °C did not amplify for *insyn2a* (H), and three samples from the bottom families at 20 °C did not amplify for *lpl*, indicating that these transcripts were not expressed in these fish at those temperatures

**Fig. 5:**
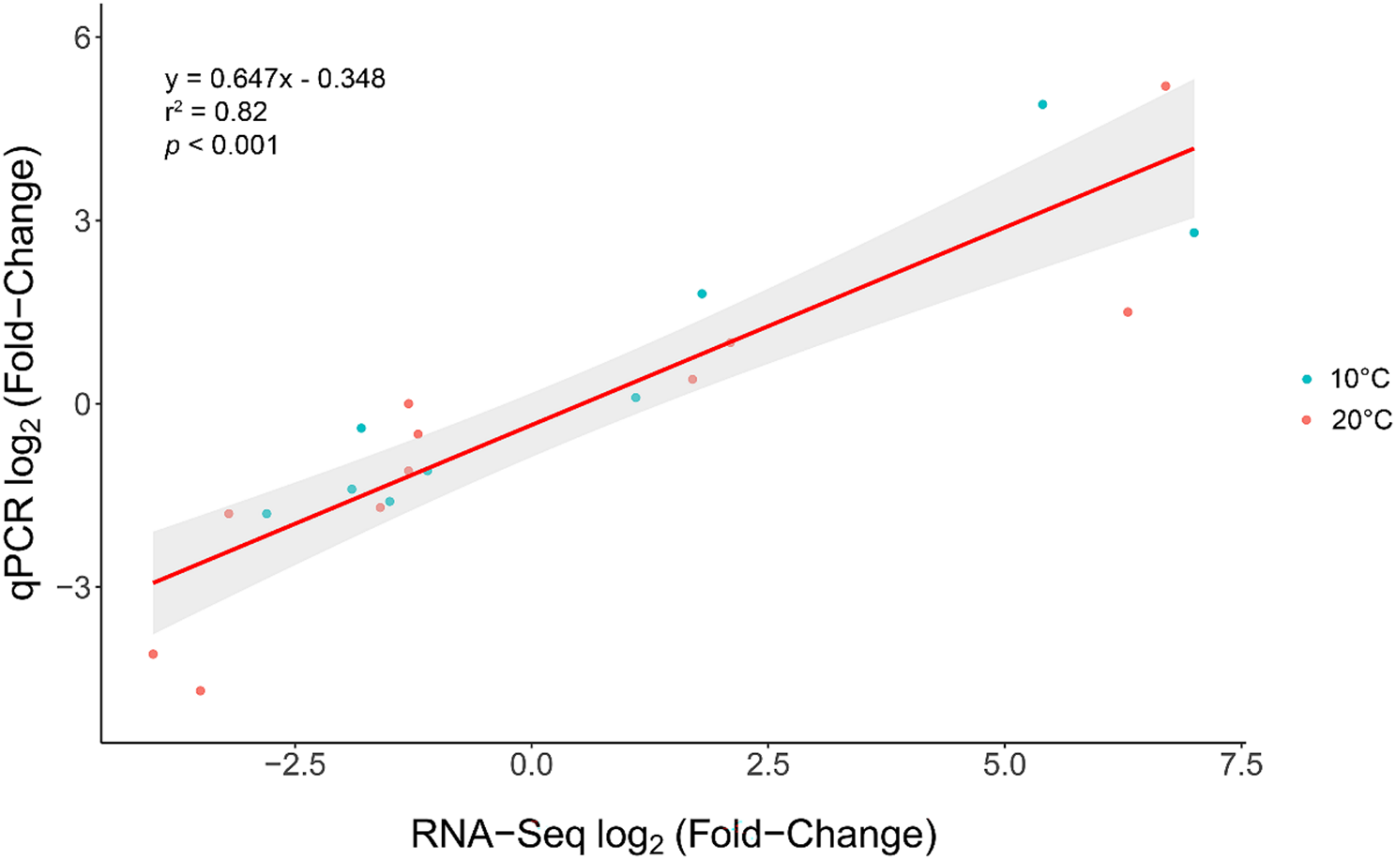
Relationship between fold-changes in transcript expression between the top and bottom thermally tolerant families at 10 and 20 °C calculated from the RNA-seq dataset (significant findings by DESeq2 only) and calibrated normalized relative quantity (CNRQ) values from the qPCR validation. The scatterplot was fitted with a linear relationship, with the shaded area surrounding it representing its standard error. The equation of the line, and the proportion of the variance attributable to the explanatory variable (r^2^), are provided. The significance of the relationship was calculated using linear modelling, and significance is indicated by the *p*-value shown

Eleven transcripts with diverse functions were chosen from the DET lists comparing the top and bottom families at 10 and 20 °C for qPCR validation (Fig. 4A-K). In almost every case, the qPCR fold-change matched the direction of change calculated by DESeq2 in the RNA-seq dataset (the exception *anxa5b* at 20 °C). This is well-reflected by a highly significant (*p* < 0.001) positive correlation (r^2^ = 0.82) between the qPCR and RNA-seq results (Fig. 5). Consequently, the RNA-seq study was determined to be successfully validated. Generally, variability was limited among a family ranking or variation was equally spread among fish within a ranking. However, *lpl* and *pdgfa* exhibited a visually apparent bimodal distribution in their expression among the top families at both temperatures. For *lpl*, F18-F20 were all represented in the top range; however, F4 was absent among the top families in that grouping. Conversely, for *pdgfa*, F4, F19 and F20 were all present in the top range, and F18 was missing. All four families within the top ranking had fish in the lower ranges for *lpl* and *pdgfa*. Therefore, there is no clear genetic basis for these distributions.

In addition, the four paralogues of *serpinh1* were assessed via qPCR (Fig. 4L-O). Near-significant (*p* < 0.08) differences (i.e., top < bottom) in *serpinh1a-1* and *serpinh1a-2* expression were detected at 10 °C. Levels of *serpinh1a-2* were significantly lower at 20 than at 10 °C in the bottom families as well. Interestingly, *serpinh1b-1* was higher expressed in the top compared to the bottom families at 10 and 20 °C. The opposite was true for *serpinh1b-2* at 10 °C. Both *serpinh1b-1* (average 23.0-fold) and *serpinh1b-2* (average 12.6-fold) were highly upregulated at 20 vs. 10 °C across the top and bottom families.

### GWAS and heritability estimates

**Fig. 6:**
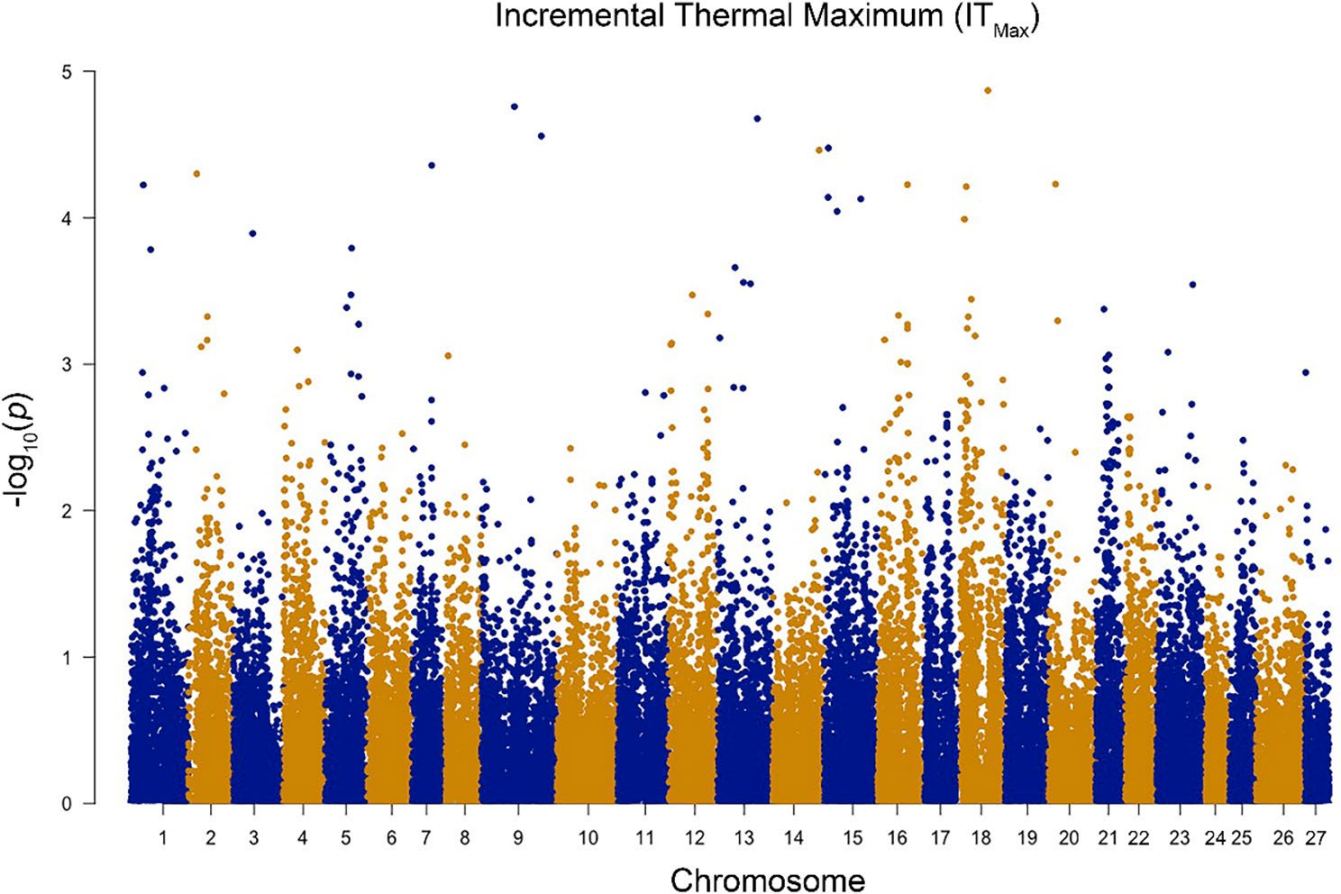
Manhattan plot of all single nucleotide polymorphisms (SNPs) associated with the incremental thermal maximum (IT_Max_) of 20 families of Atlantic salmon. No SNPs surpassed the significance threshold [-log_10_(5.0)]

**Fig. 7:**
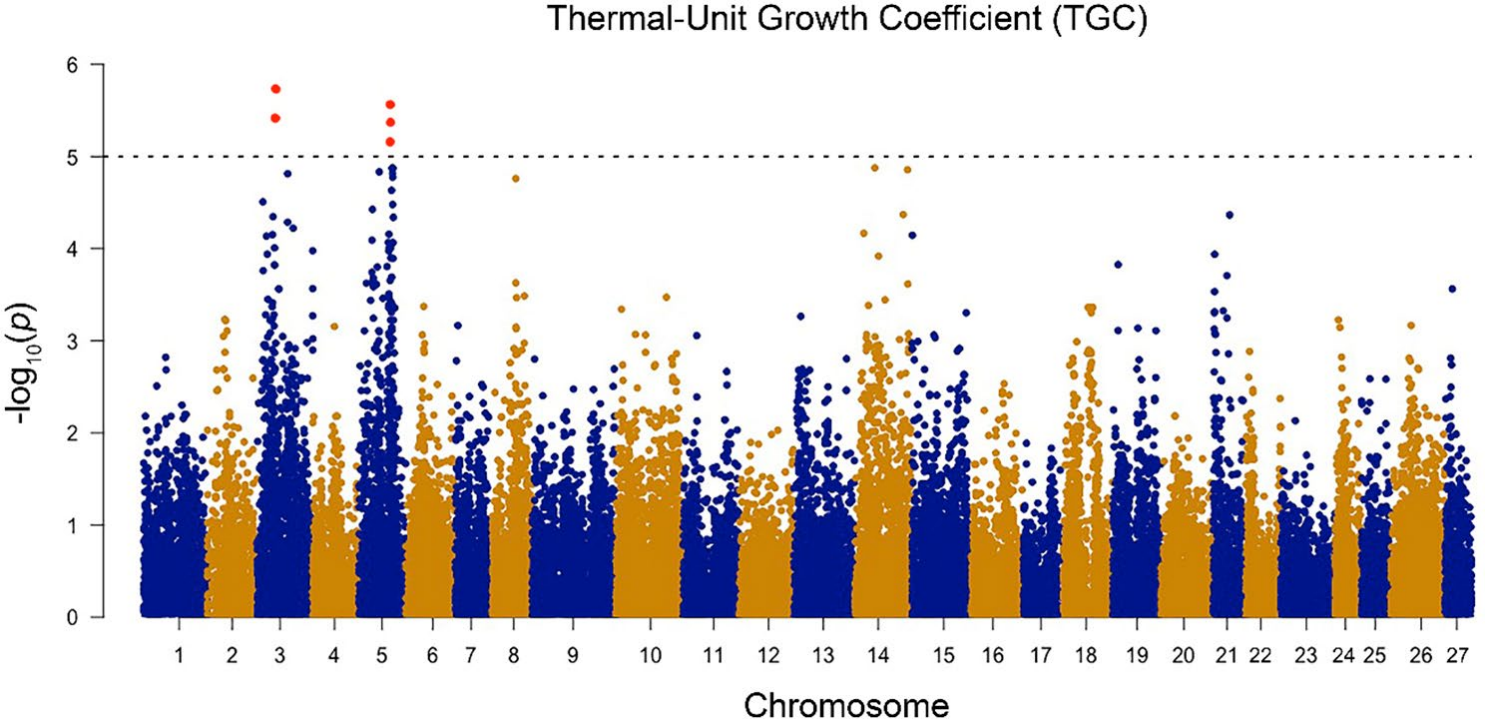
Manhattan plot of all single nucleotide polymorphisms (SNPs) associated with the thermal-unit growth coefficient (TGC) of 20 families of Atlantic salmon during an incremental thermal challenge from 12 to 20 °C. SNPs that surpassed the significance threshold [-log_10_(5.0)] are in red above the dashed line

To assess the genetic architecture of survival and growth at high temperatures in Atlantic salmon, GWAS were conducted for IT_Max_ and TGC, respectively. No SNPs surpassed the significance threshold [-log_10_(5.0)] for the highly polygenic IT_Max_ trait (Fig. 6; quantile-quantile (QQ) plot in Supplemental Figure S1). Co-localization analysis identified three DETs from the comparisons of the top and bottom families at 10 and 20 °C [i.e., *galactose-3-O-sulfotransferase 1a* (*gal3st1a*), *neurabin-1-like* (*ppp1r9a*), *coagulation factor V* (*f5*)] that were in the range (± 120 kbp) of near-significant (unadjusted *p* < 0.002) SNPs from the GWAS (Supplemental Table S12). Notably, *f5* was the highest upregulated transcript in the top vs. bottom families at 20 °C. In the TGC GWAS, five significant SNPs were detected on chromosomes three and five (Fig. 7; Supplemental Table S13; QQ plot in Supplemental Figure S2). None of the genes that these SNPs are located within or near matched any of the DETs in this study. Low/moderate heritability of IT_Max_ was estimated (SNP-based h^2^ = 0.20 ± 0.21 and pedigree-based h^2^ = 0.25 ± 0.11; mean ± SE), but high heritability estimates were calculated for TGC (SNP-based h^2^ = 0.62 ± 0.11 and pedigree-based h^2^ = 0.64 ± 0.11). A small negative genetic correlation (r^2^ = -0.07) was found between IT_Max_ and TGC. Ultimately, these results can help inform genomic breeding strategies for enhancing upper thermal tolerance and growth at high temperatures in Atlantic salmon.

## Discussion

The main aims of this study were to investigate the genetic architecture underlying IT_Max_ and TGC traits among farmed Atlantic salmon families and to compare the hepatic transcriptomic profiles of the most and least thermally tolerant families. IT_Max_ and TGC are heritable, demonstrating that these attributes can be improved through a selective breeding program. While both traits are polygenic, five significant SNPs were identified for TGC and, thus, warrant further investigation. Although no SNPs met the significance criteria in the IT_Max_ GWAS, three SNPs (*p* < 0.002) were identified to be in relative proximity to DETs from the RNA-seq experiment. Therefore, this work provides a solid foundation for future validation studies. In addition, the transcriptomic analysis identified several transcripts and pathways as possible determinants of upper thermal tolerance in Atlantic salmon. Establishing ways to proactively mitigate decreases in salmon growth and survival will be essential if the aquaculture industry is to navigate through the challenges of climate change and remain a sustainable and profitable sector. Genomics-assisted selective breeding could be one of salmon farming’s best tools to combat rising ocean temperatures.

### Transcriptomic regulation of IT_Max_

The RNA-seq analysis provided a global perspective on how Atlantic salmon families with differences in upper thermal tolerance regulate transcription at non-stressful (10 °C) and suboptimal high (20 °C) temperatures below their IT_Max_. While it must be acknowledged that RNA expression does not always equate to protein production, there is a moderate correlation between how salmonids respond to heat stress at the transcriptional level compared to the translational response [89]. Therefore, transcriptomics can be a useful preliminary tool to assess underlying mechanisms driving phenotypic traits of interest. While both sets of families responded to elevated temperature similarly, and many of the overlapping DETs also align with past research on the effects of chronic heat stress on the liver of Atlantic salmon and other fish species [35, 38, 90–92], there were still significant transcriptional differences.

For example, the DET with the highest loading on the first principal component axis, *elf3*, transactivates collagenase and *ccl20* (which had the ninth-highest loading on PC1) [93]. As *elf3* was higher expressed in the bottom families at 10 and 20 °C, this could suggest that there is a higher amount of collagen remodelling occurring in these fish. Interestingly, there was a trend (*p* < 0.08) which suggested that levels of the collagen-mediated chaperones *serpinh1a-1* and *serpinh1a-2* were higher in the bottom families compared to the top families at 10 °C. In contrast, *serpinh1b-1* was lower in the bottom families at both temperatures, and *serpinh1b-2* was higher at 10 °C. This further suggests differences in how these families regulate collagen formation and structure. Finally, although it has been suggested that small heat shock proteins and *hsp90* genes are linked to acute upper thermal tolerance in salmonids [94], none of these transcripts were differentially expressed between the top and bottom families in the current study. However, the differences in *serpinh1* expression, particularly *serpinh1b-1* (alias *hsp47b-1*), still hint that *hsp* genes have a role in regulating high temperature survival in salmon.

Interestingly, ‘sterol metabolic process’ and its downstream GO term ‘regulation of cholesterol metabolic process’ were only enriched in the top families when comparing between temperatures, while ‘regulation of cholesterol storage’ was unique to the bottom families. Previously, supplemental dietary cholesterol was not found to influence the IT_Max_ of female triploid Atlantic salmon [24]. However, increasing cholesterol in the diet did limit fillet ‘bleaching’ and potentially lowered basal cortisol levels, suggesting that it played a role in reducing organismal stress [24]. Thus, the findings in the current study support further investigation into the function of cholesterol and its relation to upper thermal tolerance in Atlantic salmon.

Another key GO term that was only enriched in the top families was ‘blood coagulation.’ The DET with the highest positive fold-change between the top and bottom families at 20 °C was *f5*, which encodes a protein putatively involved in the cleavage and activation of prothrombin into thrombin [95]. This transcript was also found within 120 kbp of a near-significant SNP from the GWAS, highlighting its potential involvement in helping determine IT_Max_ in Atlantic salmon. Blood coagulation time has been shown to decrease with rising temperatures and during CT_Max_ tests in tilapia (*Oreochromis mossambicus*; [96]). High amounts of blood clotting were reported in meagre (*Argyrosomus regius*) that perished at 34 °C [97], and blood coagulation activation was also found in humans with elevated body temperatures [98]. It is possible that changes in blood coagulation associated with thermal stress are linked to an acute phase response [99] and regulated in part by extracellular exosomal proteins in the liver [100]. This is an interesting possibility as several extracellular component GO terms were enriched when comparing the top and bottom families. Given that transcripts like *pdgfa* and *angl4* were also differentially expressed, this supports that there were likely changes in angiogenetic processes [101, 102] between the top and bottom families. Further, the hepatic expression of *rhombotin-2* (*lmo2*), another transcript with functions related to angiogenesis [103], has been linked to CT_Max_ performance in rainbow trout [92]. Altogether, these results suggest that blood coagulation and vessel formation pathways require further exploratory research, especially to determine if there is a potential association with blood oxygen transport. Although critically debated [104], the oxygen- and capacity-limited thermal tolerance (OCLTT) hypothesis [105] remains one of the leading explanations of upper thermal tolerance in aquatic species. Given that cardiac function is often incorporated into this hypothesis, and that several studies suggest its importance as a primary determinant of temperature tolerance in fish [22, 106–108], respirometry trials where cardiac function is also measured, could potentially help connect the present transcript expression results with cardiac performance measurements and haematological parameters during an IT_Max_ challenge.

### Heritability and genetic regulation of IT_Max_

Determining whether a phenotypic trait can be passed from parents to offspring, and the likelihood of that occurring, are essential first steps in breeding program management. Recognizing that the standard error values for the heritability estimates of IT_Max_ in the current study are large due to the limited sample size, these data support the findings of Gonen et al. [26] who reported similar pedigree-based (0.16 ± 0.04) and genomic-based (0.21 ± 0.04) heritabilities for IT_Max_ in a separate population of St. John River strain Atlantic salmon. However, Benfey et al. [13] estimated higher pedigree-based heritabilities for IT_Max_ in the year classes immediately before (0.40 ± 0.08) and after (0.40 ± 0.05) the one tested in Gonen et al. [26]. Therefore, it will be essential to reassess IT_Max_ across year classes in Atlantic Canadian breeding programs to accurately predict increases in the upper thermal tolerance of Atlantic salmon.

Estimates of how much incremental/chronic upper thermal tolerance is controlled by genetic variation have also been calculated in other fish species. A high mean heritability for IT_Max_ (h^2^ = 0.62) was reported for olive flounder (*Paralichthys olivaceus*) whose temperature was gradually increased to 32.5 °C from 19.4 °C over 16 days [109]. However, many studies have reported much lower heritability values for this metric of thermal tolerance. In juvenile rainbow trout exposed to chronic high temperatures (18–22 °C) for 62 days, the heritability estimate for days to death was lower at 0.13 ± 0.05 [29]. Upper thermal tolerance of killifish exposed to increases of 2°C day^-1^ had mean heritabilities of 0.22 or 0.11 based on whether they came from normal or bottlenecked lines, respectively [110]. Low heritability (mean 0.09) was also reported in turbot (*Scophthalmus maximus*) when temperature was gradually raised from 13 °C and held at 29 °C over a total period of 34 days [111]. Ultimately, these data show that there is variation in genetic selection potential among teleosts, and highlight the importance of making assessments at the species level to determine whether breeding can be an effective mitigation strategy against climate change-related increases in water temperatures.

Mean pedigree-based heritability estimates for CT_Max_ differ among Atlantic salmon (h^2^ = 0.38–0.60 dependent upon acclimation temperature - [14]; h^2^ = 0.47 - [13]), rainbow trout (h^2^ = 0.29; [16]), zebrafish (h^2^ = 0.10–0.24 dependent upon selection line; [17]), killifish (h^2^ = 0.20; [18]) and mosquitofish (h^2^ = 0.32; [19]). Experiments involving acute transfers to high temperatures where constant temperature is maintained thereafter [i.e., upper incipient lethal temperature (UILT) tests] have also been used to estimate heritability values for the time until loss of equilibrium or death in rainbow trout (h^2^ = 0.18; [112]), Lahontan cutthroat trout (*Oncorhynchus clarki henshawi*; h^2^ = 0.44; [113]), guppies (*Poecilia reticulata*; h^2^ = 0.18; [114]) and killifish (h^2^ < 0.15; [115]). After six generations of selection for improved acute upper thermal tolerance (i.e., CT_Max_), decreased thermal plasticity was reported in zebrafish, suggesting a hard upper limit for this trait [17]. Considering that no relationship between CT_Max_ and IT_Max_ has been identified in Atlantic salmon [21, 23], it would be worthwhile to test how effective genetic selection for IT_Max_ is over multiple generations. Further, as improvements in upper thermal tolerance may be small in each generation (0.04 °C; [17]), it would be advantageous to know what the rate of improvement and the upper limit of IT_Max_ could be to determine if the Atlantic salmon aquaculture sector can outpace climate change-induced increases in ocean temperatures. This is particularly relevant as in a population of Chinook salmon naturally adapted to higher temperatures, no evidence of additive genetic variation for survival at elevated temperatures was found [116], suggesting that this population had reached their thermal upper limit. Further, Penney et al. [117] provided evidence in lake trout (*Salvelinus namaycush*) that there was limited transgenerational plasticity in improving CT_Max_ in this species, and this highlights that climate change could challenge this salmonid species.

No significant SNP markers associated with IT_Max_ in Atlantic salmon were identified in the GWAS, as the trait appears to be regulated by a relatively large number of loci of small effect, as was reported previously by Gonen et al. [26]. This may be due to the fact that our limited sample size (*n* = 251) only provided us with enough power to detect large QTLs. However, even with a much larger sample size (*n* = 1532), Gonen et al. [26] did not detect any significant markers correlated with IT_Max_ in Atlantic salmon. Other studies have identified significant SNPs, QTLs and/or genes linked to upper thermal tolerance in rainbow trout [16, 118–121], cutthroat trout [122], Arctic charr [94, 123], yellow croaker (*Larimichthys crocea*; [124]), olive flounder [109] and zebrafish [125]. While none of the markers in these studies directly overlap with the three co-localized DETs (*gal3st1a*, *ppp1r9a*, *f5*) within the range of near-significant SNPs in the current study, Udayantha et al. [109] reported 66 candidate SNPs involved in thermal tolerance that fell within the category of neural/neuroendocrine function. This is noteworthy as, based on mammalian research, *gal3st1a* and *ppp1r9a* both have neurological roles [126, 127]. These results support the hypothesis that upper thermal tolerance in fish may be linked to neural activity [128, 129].

It will be important to further explore the genetic relationships between IT_Max_ and other commercially relevant traits that producers select for in breeding programs. For example, does the selection of individuals for improved upper thermal tolerance impact other environmental tolerances? In rainbow trout, no relationship was found between CT_Max_ and acute hypoxia resistance when assessed at two life stages (i.e., at 6 and 15 months old) [130]. Thus, selecting for improved acute hyperthermia tolerance may not impact the trout’s ability to withstand low oxygen conditions [130]. However, if a commercial producer wanted to improve both attributes, this would require additional phenotypic testing and selection of genetic markers of resistance, increasing the financial and logistical requirements to enhance these traits. In the current study, there was a weak negative correlation (r^2^ = -0.07) between IT_Max_ and TGC, while no phenotypic correlation was previously reported in the same fish [23]. Therefore, it is unlikely that selecting for improved IT_Max_ in Atlantic salmon would have a meaningful effect on growth at high temperatures.

### Heritability and genetic regulation of TGC

Thorland et al. [131] was the first to report heritability estimates for TGC across a production cycle of Atlantic salmon in Norway. However, the average temperatures in that trial only ranged between 8.9 and 12.4 °C [131]. Interestingly, at lower temperatures, the average pedigree-based heritability of TGC ranged between 0.35 and 0.56 [131], while the present experiment estimated higher values (mean pedigree-based h^2^ = 0.64) as salmon grew as temperatures were raised from 12 to 20 °C. The high heritability estimate for TGC in this study could be due to our limited sample size, and this hypothesis is supported by comparing our values with other studies. For example, a recent study examined TGC in Atlantic salmon grown over the Tasmanian summer and found that heritability estimates usually ranged from 0.20 to 0.40, depending upon which reaction norm model was applied [132]. In rainbow trout grown at 20 °C, the mean heritability of TGC was 0.46 [133]. Similarly, the heritability of final fish weight after rainbow trout were reared at 18–22 °C for approximately two months was 0.41 ± 0.14 [29]. Further analysis of the same trout found that the mean heritability for average daily weight gain ranged between 0.36 and 0.40 dependent upon the statistical model [134]. However, none of the genes that the five significant SNPs in the current GWAS are located within/near overlap with the SNPs identified in Yoshida and Yáñez [134] or Carvalheiro et al. [132]. Nor do our results align with those of other GWAS in Atlantic salmon that studied growth and/or weight over different production periods [135–137]. Altogether, these findings highlight that the genomic regulation of growth at high temperatures likely differs from that at lower temperatures and of other species/strains, and that Atlantic salmon genetic breeding programs should account for this if they want to improve performance during the warm summer months. Therefore, our results on TGC heritability and SNPs associated with growth at high temperatures are valuable tools for the industry in Atlantic Canada.

The five significant SNPs from the GWAS fall within or near *cyclin dependent kinase 5* (*cdk5*), *autophagy related 9B* (*atg9b*), *alpha-1*,*2-mannosidase* (*ma1a1*) and *POU class 3 homeobox 1* (*pou3f1*). Based on the mammalian literature, two of these genes (*cdk5* and *p3f3a*) have brain and/or neuron functions [138], while *atg9b* helps regulate autophagy [139] and *ma1a1* is involved in glycoprotein synthesis [140]. It is recommended that these markers in Atlantic salmon be further studied to determine if there are evolutionarily distinct functions in a salmonid that may relate more directly to growth at high temperatures. Further, validation through repeated selection in a breeding program with higher sample sizes than the current study would help confirm the effectiveness of these SNP markers in predicting TGC during the seasonal transition to summer.

### Vitellogenin

While not the focus of the present study, there were several interesting general findings from assessing *vitellogenin* in these salmon. For example, although it would require proper histological examination to validate, *vtgAsa1* appears to make for a good biomarker of sexual maturation in female Atlantic salmon at moderate (10 °C) and high (20 °C) temperatures. Levels of *vtgAsa1* were also positively correlated with the weight and hepatosomatic index (HSI) of female fish. However, these signs of early sexual maturation did not appear to influence IT_Max_ as there was no relationship between fish weight and upper thermal tolerance in either sex. However, a weak, but significant, negative correlation (r^2^ = -0.04) between IT_Max_ and HSI was found [23]. Therefore, it would be worthwhile to test whether IT_Max_ is lower in more sexually mature females, as HSI increases with reproductive development in female salmonids [141]. If so, *vtgAsa1* could potentially serve as a useful proxy biomarker of upper thermal tolerance in adult female Atlantic salmon. Future research could also explore *vitellogenin*’s immune-relevant roles, including its antioxidant activity [142], to determine if there is a connection aside from sexual maturation in relation to upper thermal tolerance in Atlantic salmon.

## Conclusion

The Atlantic salmon aquaculture industry must actively prepare for the challenges of climate change to help ensure optimal fish production and welfare. Mitigation tools are urgently needed to address rising sea surface temperatures and more frequent and severe marine heatwaves, particularly in the Northwest Atlantic [2]. Genomic selection is likely an effective way to maximize the growth and survival of salmon under these suboptimal environmental conditions. As IT_Max_ and TGC are heritable, the GWAS results are valuable for identifying candidate SNP biomarkers for validation. Of particular interest are the five significant SNPs associated with TGC, and the three near-significant co-localized SNPs linked to IT_Max_ that overlap with the RNA-seq results. The validated transcriptomic study also provides novel insights into the mechanistic drivers that determine upper thermal tolerance in Atlantic salmon. Notably, nervous system and blood coagulation processes appear to be key regulatory determinants of IT_Max_, as demonstrated by the differential expression, gene ontology enrichment and co-localization analyses. Future research into these putative biomarkers should incorporate structural genomic and epigenetic variation analyses, as recommended by Layton and Bradbury [143]. This would increase our understanding of the molecular mechanisms regulating the Atlantic salmon’s upper thermal tolerance, and potentially better predict this species’ vulnerability to climate change drivers. The future of Atlantic salmon farming relies on innovation, and further genomics research will be an essential part of sustainable expansion in a changing global environment.

## Electronic supplementary material

Below is the link to the electronic supplementary material.

Supplementary Material 1

Supplementary Material 2

Supplementary Material 3

Supplementary Material 4

Supplementary Material 5

Supplementary Material 6

Supplementary Material 7

Supplementary Material 8

Supplementary Material 9

Supplementary Material 10

Supplementary Material 11

Supplementary Material 12

Supplementary Material 13

Supplementary Material 14

Supplementary Material 15

Supplementary Material 16

Supplementary Material 17

## Data Availability

The RNA-seq dataset is available online through BioProject Accession PRJNA912749. Individual sample SRA Accessions are listed in Supplemental Table S1. All other data will be made available upon request.
