# Supplementary material for: Application of genomic tools to study and potentially improve the upper thermal tolerance of farmed Atlantic salmon (Salmo salar)
Source: BMC Genomics. 2025 Mar 24;26:294. doi: 10.1186/s12864-025-11482-4 (PMC11934803; doi:10.1186/s12864-025-11482-4)
Supplement: Supplementary file 14 — Supplementary Material 14 [file 12864_2025_11482_MOESM14_ESM.docx]

**Supplemental File S1.** RNA-sequencing methods.

After the quality of total RNA samples were confirmed, they were arrayed into a 96-well plate (Thermo Fisher Scientific). Polyadenylated (PolyA+) RNA was purified using the NEBNext Poly(A) mRNA Magnetic Isolation Module (E7490L, New England Biolabs; Whitby, ON, Canada) from 1000 ng of total RNA. Messenger RNA selection was performed using NEBNext Oligod(T)_25_ beads (NEB) incubated at 65°C for 5 min followed by snap-chilling at 4°C to denature RNA and facilitate binding of poly(A) mRNA to the beads. mRNA was eluted from the beads in Tris Buffer incubated at 80°C for 2 min then held at 25°C for 2 min. RNA binding buffer was added to allow the mRNA to re-bind to the beads, mixed, and incubated at room temperature for 5 min. The sample plate was placed on a magnet and the supernatant was discarded. The mRNA bound beads were washed twice, and the supernatant was again discarded. mRNA was eluted from the beads in 20 µL of Tris buffer following incubation at 80°C for 2 min. mRNA was then transferred to a new 96-well plate.

First-strand cDNA was synthesized from heat-denatured purified mRNA using a Maxima H Minus First Strand cDNA Synthesis kit (Thermo Fisher Scientific) and random hexamer primers at a concentration of 200 ng µL^-1^ along with Actinomycin D (40 ng µL^-1^), followed by PCR Clean DX (Aline Biosciences; Waltham, MA, USA) bead purification on a Microlab NIMBUS robot (Hamilton; Reno, NV, USA). The second strand cDNA was synthesized following the NEBNext Ultra Directional Second Strand cDNA Synthesis protocol (New England Biolabs) that incorporates dUTP in the dNTP mix, allowing the second strand to be digested using the USER™ enzyme (NEB) in the post-adapter ligation reaction and thus achieving strand specificity.

cDNAs were fragmented by Covaris LE220 sonication to achieve 250-300 bp average fragment lengths. The paired-end sequencing library was prepared following the BC Cancer Genome Sciences Centre strand-specific, plate-based library construction protocol on a Microlab NIMBUS robot. Briefly, the sheared cDNA was subject to end-repair and phosphorylation in a single reaction using an enzyme premix (New England Biolabs) containing T4 DNA polymerase, Klenow DNA Polymerase and T4 polynucleotide kinase, incubated at 20°C for 30 min. Repaired cDNA was purified in a 96-well format using PCR Clean DX beads, and 3’ A-tailed (adenylation) using Klenow fragment (3’ to 5’ exo minus) with incubation at 37°C for 30 min prior to enzyme heat inactivation. Illumina TruSeq adapters were ligated at 20°C for 15 min. The adapter-ligated products were purified using PCR Clean DX beads, then digested with USER™ enzyme (1 U µL^-1^, NEB) at 37°C for 15 min followed immediately by 10 cycles of indexed PCR using NEBNext Ultra II Q5 Master Mix (New England Biolabs) and Illumina’s primer set. PCR parameters were: 1 cycle of 98°C for 1 min, 10 cycles of 98°C for 15 sec, 65°C for 30 sec and 72°C for 30 sec, and then 1 cycle of 72°C for 5 min. The PCR products were purified and size-selected using a 1:1 PCR Clean DX beads-to-sample ratio (twice), and the eluted DNA quality was assessed with Caliper LabChip GX for DNA samples using the High Sensitivity Assay (PerkinElmer, Inc.; Waltham, MA, USA) and quantified using a Quant-iT dsDNA High Sensitivity Assay Kit on a Qubit fluorometer (Invitrogen) prior to library pooling and size-corrected final molar concentration calculation for Illumina sequencing with paired-end 150 bp reads. A total of 93 stranded paired-end 150 bp libraries were created from the RNA samples collected from the top and bottom thermally tolerant families at 10 and 20°C using an Illumina NovaSeq 6000 platform (v.1.5 chemistry) targeting 50M reads per library.
