# Supplementary material for: Application of genomic tools to study and potentially improve the upper thermal tolerance of farmed Atlantic salmon (Salmo salar)
Source: BMC Genomics. 2025 Mar 24;26:294. doi: 10.1186/s12864-025-11482-4 (PMC11934803; doi:10.1186/s12864-025-11482-4)
Supplement: Supplementary file 16 — Supplementary Material 16 [file 12864_2025_11482_MOESM16_ESM.pdf]

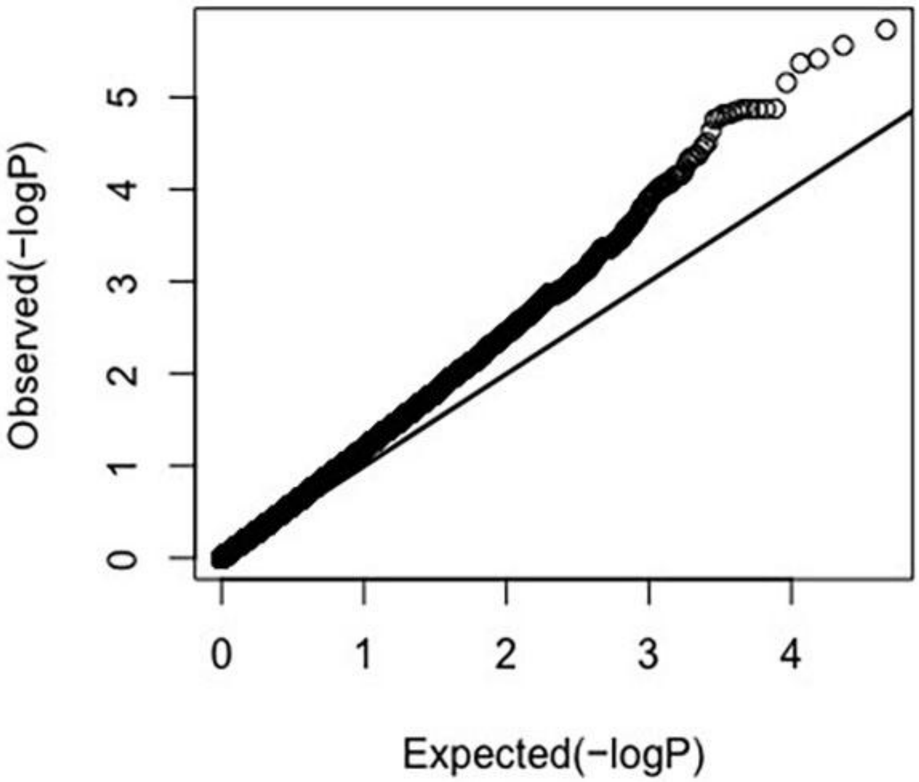

Supplemental Figure S2. Quantile-quantile (QQ) plot of the observed versus expected *p*-values from the thermal-unit growth coefficient (TGC) Genome-Wide Association Study (GWAS).
