# Supplementary material for: Application of genomic tools to study and potentially improve the upper thermal tolerance of farmed Atlantic salmon (Salmo salar)
Source: BMC Genomics. 2025 Mar 24;26:294. doi: 10.1186/s12864-025-11482-4 (PMC11934803; doi:10.1186/s12864-025-11482-4)
Supplement: Supplementary file 17 — Supplementary Material 17 [file 12864_2025_11482_MOESM17_ESM.docx]

To determine whether sexual maturation could be a potential confounding factor in the present study, levels of *vitellogenin* (*vtgAsa1*) were measured by qPCR before samples were RNA-sequenced. In female Atlantic salmon, *vtgAsa1* expression was higher in bottom-ranked compared to the top thermally tolerant families at 10 and 20°C (Figure S2-1A). However, this pattern was not visually consistent among families (Figure S2-1B). For male salmon, no difference in the expression of *vtgAsa1* was found between family rankings (Figure S2-2A), and similarly, no pattern was evident among individual families at either temperature (Figure S2-2B). Regardless of family ranking, levels of *vtgAsa1* were higher in females than males at both temperatures (Figure S2-3A). When data were pooled between sexes, *vtgAsa1* expression was higher in the bottom compared to the top families at 10 and 20°C (Figure S2-3B). Yet, this seems to be primarily driven by F5 and F6, particularly at 10°C (Figure S2-3C). Among the top and bottom families, levels of *vtgAsa1* were positively correlated with fish weight (Figure S2-4A) and hepatosomatic index (HSI; Figure S2-4B) in female salmon. These relationships were both absent in male fish though (Figure S2-5A,B). In salmon that reached their incremental thermal maximum (IT_Max_) from the top and bottom families (i.e., separate from the fish that were sampled for liver transcript expression), no correlations were found between fish weight and IT_Max_ in female (Figure S2-6A), male (Figure S2-6B) or mixed-sex (Figure S2-6C) comparisons. Ignatz et al. [23] already showed that there was no relationship between weight and IT_Max_ across all 20 mixed-sex families in that experiment, but this was also true when female (Figure S2-7A) and male (Figure S2-7B) salmon were assessed separately. Collectively, these results show that while some female Atlantic salmon were sexually maturing during the study, this was unlikely to have impacted their upper thermal tolerance. As a precaution though, all *vitellogenin* transcripts and any transcripts that were hierarchically clustered and significantly correlated with *vitellogenin* expression were removed from downstream analyses (Supplemental Table S2). Therefore, we are confident that the final lists of differentially expressed transcripts (DETs) reported accurately represent how the top and bottom thermally tolerant families distinctly respond to high temperature stress.

T-tests were used when comparisons were only made between two variables (i.e., comparisons between family rankings or sexes). Linear mixed-effect models were used to examine if weight or HSI were related to *vitellogenin* expression and/or IT_Max_, using family and tank as fixed and random factors, respectively. All statistical procedures were carried out in R (v. 4.1.2) and a difference was considered significant if *p* < 0.05.


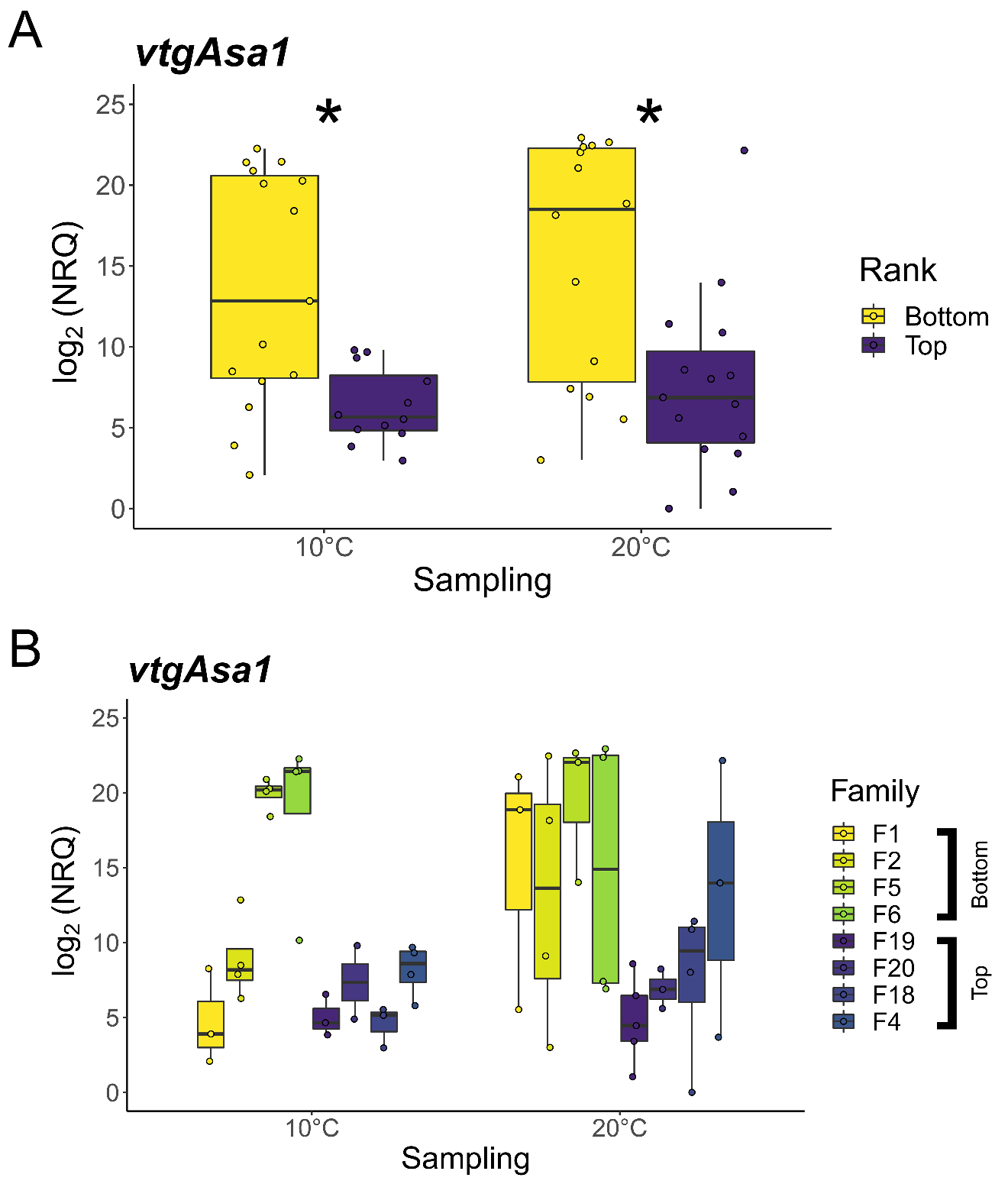


**Supplemental File Figure S2-1.** Transcript expression levels of *vitellogenin* (*vtgAsa1*) in the liver of female Atlantic salmon sampled at either 10 or 20°C across the 4 bottom and 4 top thermally tolerant families. A) Expression of *vtgAsa1* pooled by family ranking (n = 12-15 per ranking at a given temperature). Normalized relative quantities (NRQs) were compared by t-tests (*p* < 0.05) between rankings at the same sampling point. Asterisks denote significant differences between rankings. B) Expression of *vtgAsa1* within each family (n = 2-5 per family at a given temperature). No statistics were performed due to low sample sizes within families.


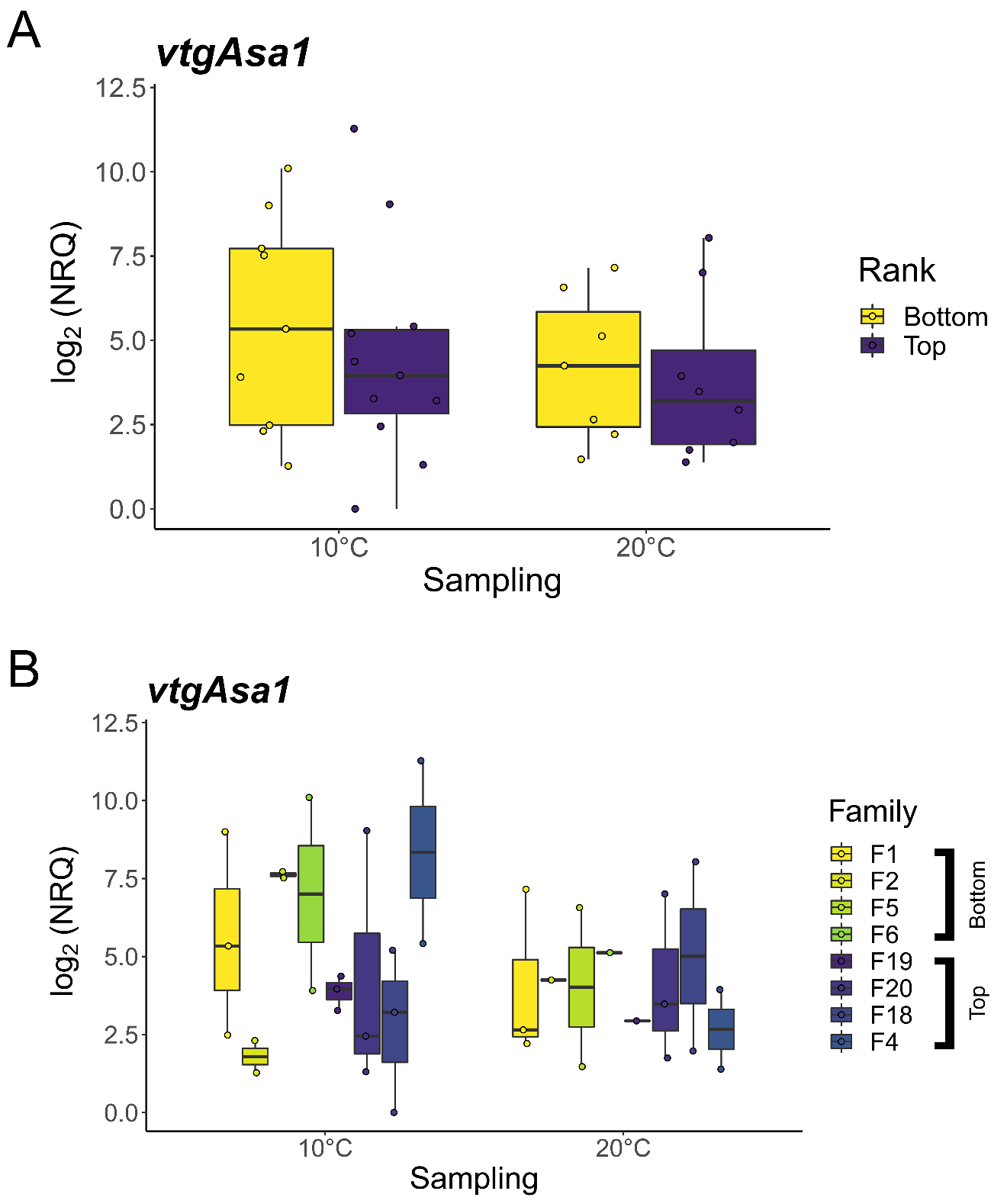


**Supplemental File Figure S2-2.** Transcript expression levels of *vitellogenin* (*vtgAsa1*) in the liver of male Atlantic salmon sampled at either 10 or 20°C across the 4 bottom and 4 top thermally tolerant families. A) Expression of *vtgAsa1* pooled by family ranking (n = 7-11 per ranking at a given temperature). Normalized relative quantities (NRQs) were compared by t-tests (*p* < 0.05) between rankings at the same sampling point. Asterisks denote significant differences between rankings. B) Expression of *vtgAsa1* within each family (n = 1-3 per family at a given temperature). No statistics were performed due to low sample sizes within families.


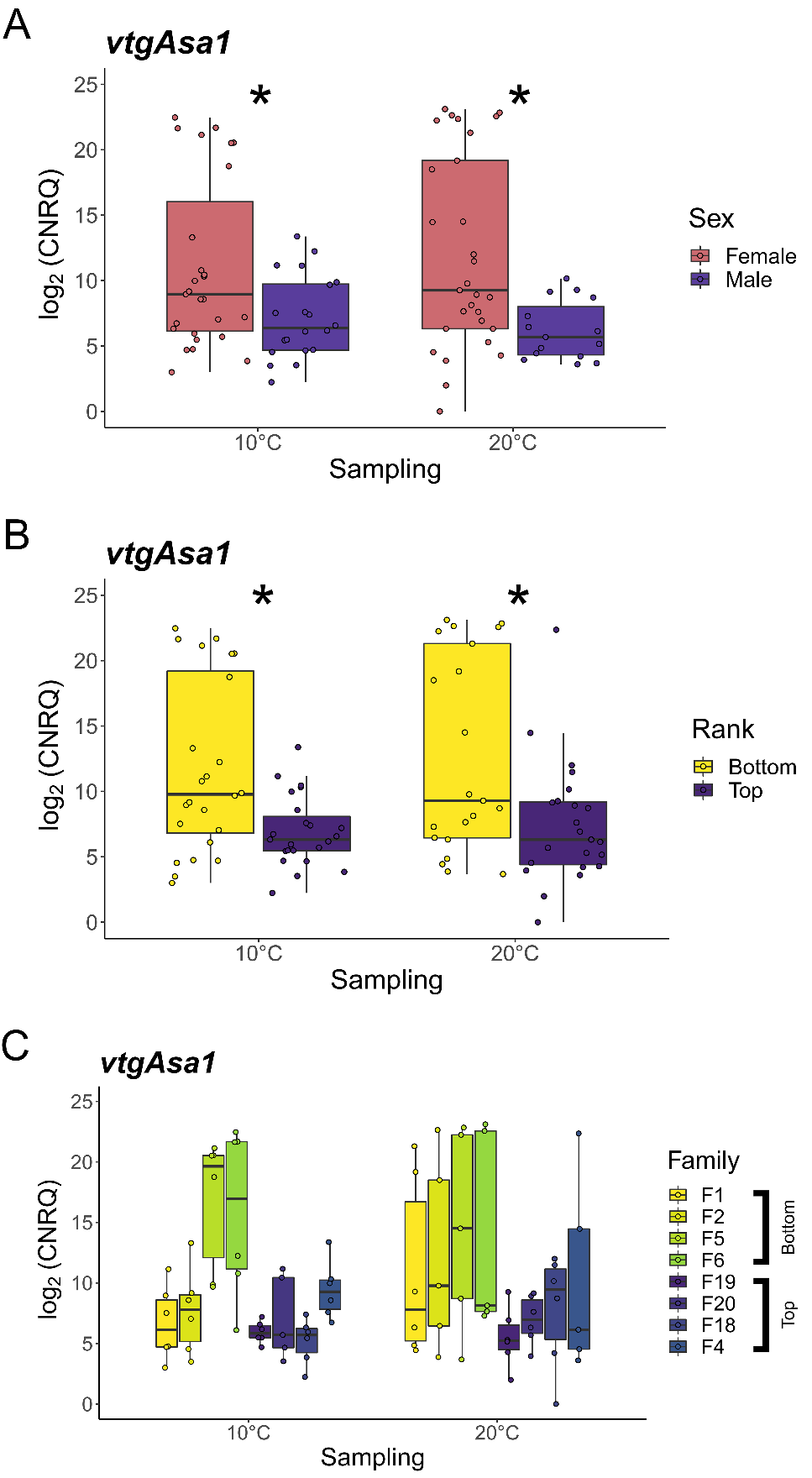


**Supplemental File Figure S2-3.** Transcript expression levels of *vitellogenin* (*vtgAsa1*) in the liver of female and male Atlantic salmon sampled at either 10 or 20°C across the 4 bottom and 4 top thermally tolerant families. A) Expression of *vtgAsa1* pooled by sex (n = 15-27 per sex at a given temperature). Calibrated normalized relative quantities (CNRQs) were compared by t-tests (*p* < 0.05) between sexes at the same sampling point. Asterisks denote significant differences between sexes. B) Expression of *vtgAsa1* pooled by family ranking (n = 21-24 per ranking at a given temperature). Calibrated normalized relative quantities (CNRQs) were compared by t-tests (*p* < 0.05) between rankings at the same sampling point. Asterisks denote significant differences between rankings. C) Expression of *vtgAsa1* within each family (n = 5-6 per family at a given temperature). No statistics were performed.


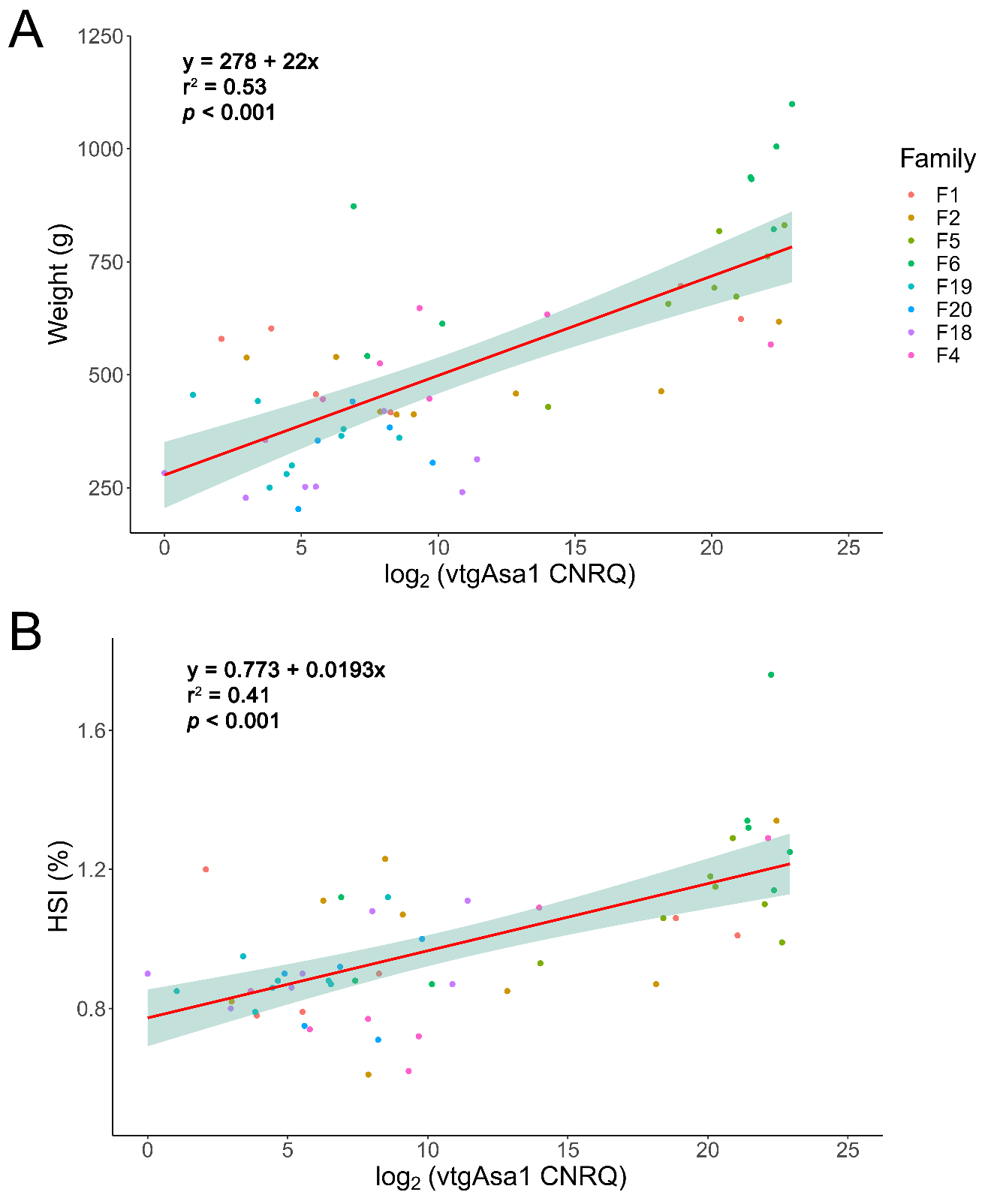


**Supplemental File Figure S2-4.** Relationships between *vitellogenin* (*vtgAsa1*) expression and A) fish weight and B) hepatosomatic index (HSI) in female Atlantic salmon (n = 56 among top and bottom families). Each scatterplot was fitted with a linear relationship, with the shaded area surrounding it representing its standard error. The equation of the line, and the proportion of the variance attributable to the explanatory variable (r^2^), are provided. The significance of the relationship was calculated using linear mixed-effect modeling, and is indicated by the *p*-value shown.


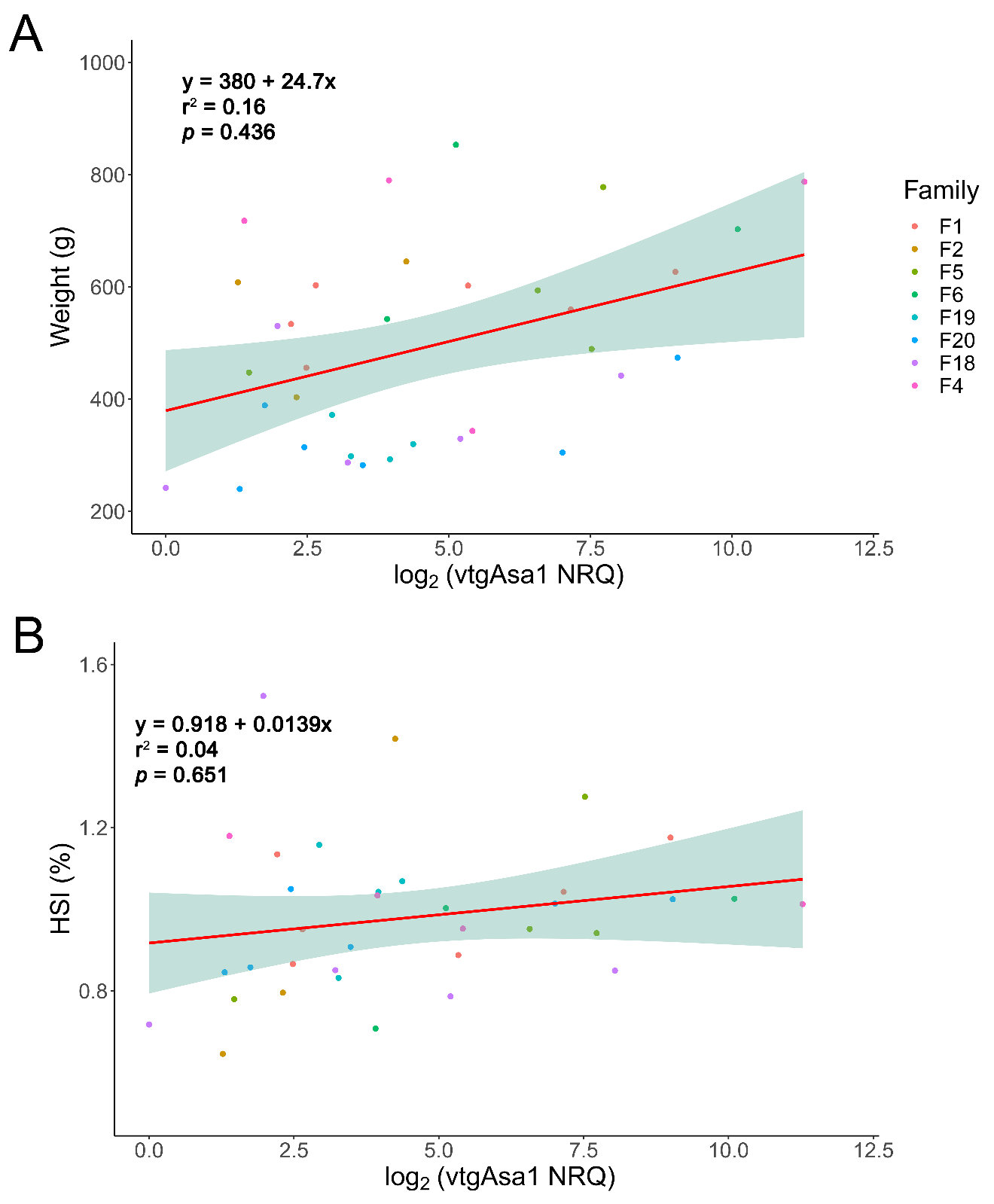


**Supplemental File Figure S2-5.** Relationships between *vitellogenin* (*vtgAsa1*) expression and A) fish weight and B) hepatosomatic index (HSI) in male Atlantic salmon (n = 35 among top and bottom families). Each scatterplot was fitted with a linear relationship, with the shaded area surrounding it representing its standard error. The equation of the line, and the proportion of the variance attributable to the explanatory variable (r^2^), are provided. The significance of the relationship was calculated using linear mixed-effect modeling, and is indicated by the *p*-value shown.


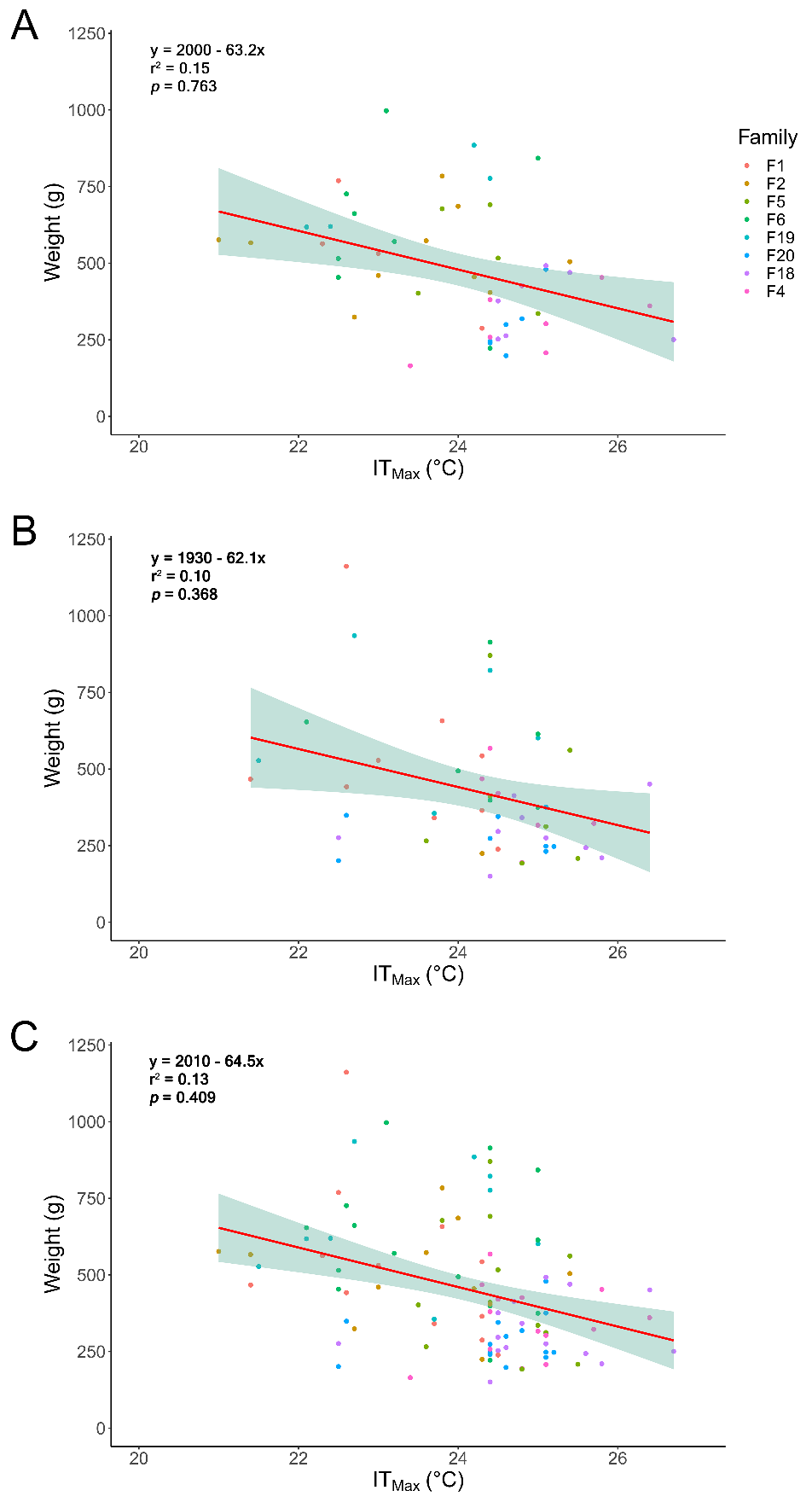


**Supplemental File Figure S2-6.** Relationships between incremental thermal maximum (IT_Max_) and fish weight in A) female (n = 51), B) male (n = 51) and C) mixed sex (n = 102) Atlantic salmon among top and bottom families. Each scatterplot was fitted with a linear relationship, with the shaded area surrounding it representing its standard error. The equation of the line, and the proportion of the variance attributable to the explanatory variable (r^2^), are provided. The significance of the relationship was calculated using linear mixed-effect modeling, and is indicated by the *p*-value shown. Data originated from Ignatz et al. [23].


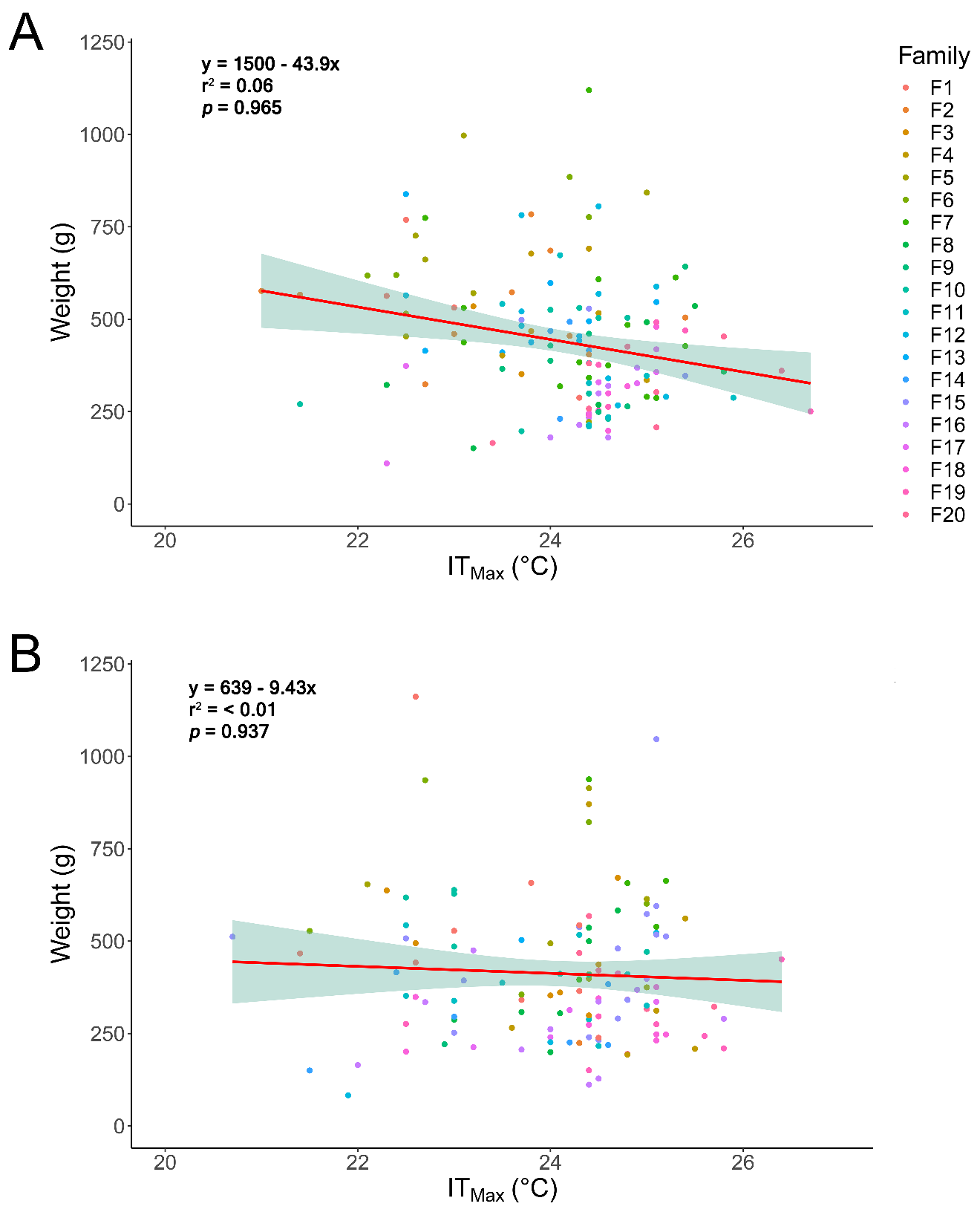


**Supplemental File Figure S2-7.** Relationships between incremental thermal maximum (IT_Max_) and fish weight in A) female (n = 139) and B) male (n = 127) Atlantic salmon among all families. Each scatterplot was fitted with a linear relationship, with the shaded area surrounding it representing its standard error. The equation of the line, and the proportion of the variance attributable to the explanatory variable (r^2^), are provided. The significance of the relationship was calculated using linear mixed-effect modeling, and is indicated by the *p*-value shown. Data originated from Ignatz et al. [23].
